# Time-Restricted Feeding Could Not Reduce Rainbow Trout Lipid Deposition Induced by Artificial Night Light

**DOI:** 10.3390/metabo12100904

**Published:** 2022-09-26

**Authors:** Hanying Xu, Ce Shi, Yangfang Ye, Changbin Song, Changkao Mu, Chunlin Wang

**Affiliations:** 1 Key Laboratory of Aquacultural Biotechnology, Ministry of Education, Ningbo University, 818 Fenghua Road, Ningbo 315211, China; 2 Marine Economic Research Center, Dong Hai Strategic Research Institute, Ningbo University, 818 Fenghua Road, Ningbo 315211, China; 3 Collaborative Innovation Center for Zhejiang Marine High-Efficiency and Healthy Aquaculture, 818 Fenghua Road, Ningbo 315211, China; 4 Institute of Semiconductors, Chinese Academy of Sciences, Beijing 100083, China

**Keywords:** artificial night light, feeding regime, lipid metabolism, serum metabolites, rainbow trout

## Abstract

Artificial night light (ALAN) could lead to circadian rhythm disorders and disrupt normal lipid metabolism, while time-restricted feeding (TRF) could maintain metabolic homeostasis. In mammals, TRF has been demonstrated to have extraordinary effects on the metabolic regulation caused by circadian rhythm disorders, but studies in lower vertebrates such as fish are still scarce. In this study, the impacts of ALAN on the body composition and lipid metabolism of juvenile rainbow trout were investigated by continuous light (LL) exposure as well as whether TRF could alleviate the negative effects of LL. The results showed that LL upregulated the expression of lipid synthesis (*fas* and *srebp-1c*) genes and suppressed the expression of lipid lipolysis (*pparβ*, *cpt-1a*, and *lpl*) genes in the liver, finally promoting lipid accumulation in juvenile rainbow trout. However, LL downregulated the expression of genes (*Δ6-fad*, *Δ9-fad*, *elovl2*, and *elovl5*) related to long-chain polyunsaturated fatty acid (LC-PUFA) synthesis, resulting in a significant decrease in the proportion of LC-PUFA in the dorsal muscle. In serum, LL led to a decrease in glucose (Glu) levels and an increase in triglyceride (TG) and high-density lipoprotein cholesterol (H-DLC) levels. On the other hand, TRF (mid-dark stage feeding (D)) and mid-light stage feeding (L)) upregulated the expression of both the lipid synthesis (*srebp-1c* and *pparγ*), lipolysis (*pparα*, *pparβ*, and *cpt-1a*), and lipid transport (*cd36*/*fat* and *fatp-1*) genes, finally increasing the whole-body lipid, liver protein, and lipid content. Meanwhile, TRF (D and L groups) increased the proportion of polyunsaturated fatty acid (PUFA) and LC-PUFA in serum. In contrast, random feeding (R group) increased the serum Glu levels and decreased TG, total cholesterol (T-CHO), and H-DLC levels, suggesting stress and poor nutritional status. In conclusion, ALAN led to lipid accumulation and a significant decrease in muscle LC-PUFA proportion, and TRF failed to rescue these negative effects.

## 1. Introduction

Light is an essential environmental factor that affects the various life activities of organisms on Earth. With the advent and large-scale application of artificial light sources, artificial light at night (ALAN) has been recognized as one of the fastest-growing factors altering the natural environment and is considered as a pollutant called light pollution [1]. Light pollution has been shown to negatively affect human society and natural ecology [1,2,3]. For living organisms, the light–dark cycle (photoperiod) is the dominant zeitgeber that guides and synchronizes circadian rhythms [4]. Circadian rhythms play an important role in maintaining energy homeostasis, and many genes and enzymes related to nutrient metabolism exhibit strong circadian rhythms [5]. ALAN breaks the normal photoperiod and disrupts the circadian rhythm of the organism. In animal models and human studies, nocturnal light exposure has been associated with metabolic disorders, leading to an increased risk of metabolic diseases such as obesity [6] and type 2 diabetes [7]. In addition to directly affecting metabolic processes through biological clock rhythms, nocturnal light exposure also affects metabolic function by suppressing melatonin, altering glucocorticoids, and changing sleep architecture [8,9]. Metabolic disruptions caused by light pollution are of increasing concern, not only with regard to human health concerns, but also for the growth, survival, and welfare of farmed animals.

Lipids are crucial nutrients that can provide energy, essential fatty acids, phospholipids, sterols, and other substances for the life activities of organisms [10,11]. For carnivorous fish, lipids are preferred energy providers due to the limited ability to utilize carbohydrates [12,13]. Maintaining normal lipid metabolic processes is the basis for the growth and development of carnivorous fish. Briefly, lipid metabolism is the process of lipid uptake and transport, synthesis, and catabolism, in which various transcription factors and enzymes are involved [14,15]. The de novo synthesis of fatty acids begins with the synthesis of acetyl coenzyme A into palmitic acid (C16:0) and stearic acid (C18:0) in the presence of fatty acid synthase (FAS), followed by the further synthesis of polyunsaturated fatty acids (PUFA) in the presence of desaturases (e.g., Δ6-fatty acid desaturase (Δ6-fad) and Δ9-fatty acid desaturase (Δ9-fad)) and elongases (e.g., elongation of very long-chain fatty acid protein 2 (elovl2) and elongation of very long-chain fatty acid protein 5 (elovl5)) [15]. The *fas* gene expression is regulated by the upstream transcription factor sterol regulatory element binding protein 1c (SREBP-1c) [16,17]. The process of lipid oxidative catabolism begins with the hydrolysis of triglycerides to fatty acids and monoacylglycerols by the action of lipoprotein lipase (LPL) [18,19]. Further oxidation of fatty acids for energy supply involves the transport of long-chain fatty acids in mitochondria, and in the liver, carnitine palmitoyltransferase 1a (cpt-1a) is the key rate-limiting enzyme [15,20]. During lipid uptake and transport, cluster of differentiation 36/fatty acid translocase (CD36/FAT) [21] and fatty acid transport protein 1 (FATP-1) [22] play essential roles. In addition, peroxisome proliferator-activated receptors (PPARs, e.g., PPARα, PPARβ, PPARγ) play a crucial role in regulating lipid metabolic processes [23,24].

Feeding/meal time can directly activate nutrient-sensing pathways to regulate metabolic processes in organisms and is independent of photoperiod [25,26]. Time-restricted feeding (TRF) is thought to prevent and treat metabolic diseases by maintaining optimal nutrient utilization [25]. In rodents, TRF was found to reduce the total cholesterol, triglyceride, glucose, and insulin levels and improve glucose control and insulin sensitivity [27]. Furthermore, in some metabolic disorder animal models and populations suffering from metabolic diseases such as in mice fed high-fat diets, TRF reduced the body fat accumulation, improved glucose tolerance, and stabilized the circadian rhythm of the central biological clock compared to ad libitum feeding [28,29,30]. In a biological clock-deficient mouse model, TRF prevented the development of obesity and metabolic syndrome [31]; TRF restored muscle function in a *Drosophila* model of obesity and rhythm disorders [32]. In overweight patients with type 2 diabetes, TRF improved glucose and insulin sensitivity [33]. In addition, in mice, TRF repaired the attenuation of biological clock rhythms in peripheral tissues (liver and white adipose tissue) due to continuous light (LL) [34]. In recent years, several review publications have summarized the positive role of TRF in regulating metabolic homeostasis [26,35,36].

Although TRF has shown extraordinary effects on metabolic regulation in humans and some model organisms, studies in lower vertebrates such as fish are still scarce [37]. Aquaculture is currently the fastest-growing form of food production globally, providing more than 82 million tons of high-quality protein for humans in 2018 [38]. Although there have been several reports focusing on the impact of light pollution on aquatic organisms [39,40,41], the negative impacts of light pollution on aquatic animals have not received much attention compared to terrestrial animals [42]. In fish, light pollution research has focused on behavior [43,44,45], community structure [46], physiology [47,48], and fitness [49], with very limited information on metabolism [50,51]. The vast majority of studies have reported that fish melatonin levels were suppressed by ALAN [42,48,52]. Melatonin is an important mediator of the conversion of exogenous temporal signals into endogenous biological rhythms [53], and the disruption of melatonin rhythms in fish by ALAN can also have an impact on lipid metabolism. Previous studies have reported that LL exposure led to an increased lipid content in some fish such as Atlantic salmon (*Salmo salar*) [54,55] and gibel carp (*Carassius auratus*) [50], which may be attributed to disturbed lipid metabolism under LL.

Rainbow trout (*Oncorhynchus mykiss*) is an economically important cold-water fish. As an important representative species of salmonids, it occupies an essential position in aquaculture production, with global aquaculture production reaching 848,051 tons and a value of 3.88 billion USD in 2018 [56]. Rainbow trout is also an important model fish, and there have been several studies on the effects of photoperiod and restricted feeding on metabolism [57,58]. As a representative species of teleost fish, it is necessary to determine the impact of light pollution on its lipid metabolism, and whether TRF can mitigate this state. Thus, this study investigated (1) the effects of ALAN on the body composition and lipid metabolism of juvenile rainbow trout by simulating light pollution with continuous light (LL) and (2) whether TRF could mitigate the negative effects of light pollution through three feeding regimes.

## 2. Materials and Methods

### 2.1. Fish

This study was approved by the Animal Care and Use Committee of Ningbo University and carried out in the recirculating aquaculture system (RAS) (HISHING, Qingdao, China) at the School of Marine Sciences pilot plant, Ningbo University from July to November 2020. The juvenile rainbow trout were purchased from a commercial nursery (Shandong, China). Fish were acclimatized for one month in the RAS and randomly fed with a commercial diet (about 2% body weight, Tech-Bank, Ningbo, China, Supplementary Table S1). The feeding schedule was provided by random number generator software (RAND function of Microsoft Excel) according to Nisembaum et al. (2012) [59]. The environmental conditions were maintained as follows: the photoperiod was 12L:12D (lights-on at 6:00, the light intensity on the water surface is 100–200 lx), the water temperature at 16.5 ℃ ± 1 °C, the dissolved oxygen was higher than 9 mg/L, and the ammonia nitrogen was lower than 0.05 mg/L.

### 2.2. Experiment Design

After the acclimation, 840 healthy fish (18.98 ± 1.69 g/fish) were weighed and randomly assigned to 42 culture tanks (volume 600 L, Supplementary Figure S1), with 20 fish in each tank. The experiment included two photoperiods: 24L:0D (LL, constant light with 100–200 lx light intensity on the water surface) and 12L:12D (LD, lights-on at 6:00, light-off at 18:00, the light intensity on the water surface is 100–200 lx); three feeding regimes: random feeding (R), mid-dark stage feeding (D), and mid-light stage feeding (L). In this study, automatic feeding machines (YF-9258, Fish Baby, Sichuan, China) were used, and the feeding time and quantity were set in advance. A total of six experimental treatments were combined: R-LL, D-LL, L-LL, R-LD, D-LD, and L-LD, and each treatment included seven culture tanks (each treatment included seven replicates). The growth experiment lasted three months, and the cultural management and environmental conditions were consistent with the acclimation.

### 2.3. Sample Collection

At the end of the three-month growth experiment, all fish were deprived of food for 24 h. To reduce the effect of food anticipatory activity (FAA) on the blood metabolite levels, sampling was started 4 h after the feeding point. That is, all fish were starved for 28 h. MS-222 was used to anesthetize the fish, and 12 fish were randomly selected from each tank for sample collection. Two fish were randomly selected and frozen at −20 °C to determine the body composition. The blood was immediately collected from the remaining 10 fish by the tail vein method. The blood was stored in a 1.5 mL EP tube, left at 4 °C overnight, and then centrifuged at 2500 rpm at 4 °C for 10 min. The supernatant (serum) was stored at −80 °C until analysis of the hematological parameters. The fish was immediately dissected after the blood was taken; the liver, intestine, and dorsal muscle were separated and quick-frozen in liquid nitrogen, then stored at −80 °C until analysis.

### 2.4. Biochemical Analysis

The biochemical composition of the whole fish, dorsal muscle, and liver was determined according to AOAC (1995) [60]. First, the sample was dried to constant weight by a freeze dryer (LL1500, Thermo Scientific, Waltham, MA, USA), and the reduced mass was the moisture; then, the crude protein content in the sample was determined by a Kjeldahl analyzer (K355/K437, Buchi, Flawil, Switzerland) and the crude lipid in the sample was determined by a Soxhlet extractor (E816, Buchi, Flawil, Switzerland). The ash content of the whole fish was determined by a muffle furnace at 550 °C for 12 h. Total protein (TP), glucose (Glu), triglyceride (TG), lactic acid (LA), total cholesterol (T-CHO), high-density lipoprotein cholesterol (H-DLC), and low-density lipoprotein cholesterol (L-DLC) in serum and glycogen in the muscle and liver were all measured using commercial kits (Nanjing Jiancheng Bioengineering Institute, Nanjing, China) according to the instructions. Fatty acids in the liver, dorsal muscle, and serum were determined by gas chromatography (GC7890B, Agilent Technologies, Santa Clara, CA, USA) as described in Liu et al. (2021) [61].

### 2.5. Gene Expression

The total RNA of the liver was extracted with a commercial kit RNA isolator (R401-01, Vazyme, Nanjing, China). The quality of total RNA was checked by an ultramicro-spectrophotometer (Nanodrop 2000, Thermo Scientific, Waltham, MA, USA) and 1% gel electrophoresis. The RNA was reverse transcribed into cDNA using a HiFiScript cDNA Synthesis Kit (CW2569M, CWBIO, Beijing, China).

Real-time PCR was used to analyze the relative expression of the lipid metabolism-related genes (fatty acid synthase (*fas*), Δ6-fatty acid desaturase (*Δ6-fad*), Δ9-fatty acid desaturase (*Δ9-fad*), elongation of very long-chain fatty acid protein 2 (elovl2), elongation of very long-chain fatty acid protein 5 (*elovl5*), sterol regulatory element binding protein 1c (*srebp1c*), peroxisome proliferators-activated receptor α (*pparα*), peroxisome proliferators-activated receptor β (*pparβ*), peroxisome proliferators-activated receptor γ (*pparγ*), carnitine palmitoyl transferase 1a (*cpt1a*), lipoprotein lipase (*lpl*), cluster of differentiation 36/fatty acid translocase (*cd36*/*fat*), and fatty acid transport protein 1 (*fatp1*)) in the liver. The total reaction volume was 20 μL including 10 μL of 2 × MagicSYBR Mixture (CW3008H, CWBIO, Beijing, China), 2 μL of cDNA, 0.4 μL of each primer (10 μM), and 7.2 μL of ddH_2_O. A Real-Time PCR System (QuantStudio™ 6 Flex, Life Technologies, Carlsbad, CA, USA) was used with the program as follows: 95 °C for 30 s; 45 cycles at 95 °C for 5 s, 60 °C for 30 s; and 95 °C for 15 s. The specific primers used in this study are shown in Supplementary Table S2 and were synthesized by a commercial company (Youkang Biological Technology Co., Ltd., Hangzhou, China). The relative expression level of the target genes was normalized by *β-actin* and elongation factor-1α (*ef1α*) and calculated by the comparative CT method (2^−ΔΔCT^ method) [62].

### 2.6. Statistical Analysis

All statistical analyses were performed on SPSS 22.0 and R 4.1.2 software. First, all data were checked for homogeneity and normal distribution through Levene’s test and the Kolmogorov–Smirnov test, respectively. Then, a two-way ANOVA was performed with the photoperiod and feeding regime. Meanwhile, t-tests were performed for the photoperiod, and one-way ANOVA followed by Duncan’s multiple range test were performed for the feeding regime. *p* < 0.05 and *p* < 0.01 were considered as significant differences and extremely significant differences, respectively. In addition, the principal component analysis (PCA) was used to analyze the liver, dorsal muscle, and serum fatty acid profile. Finally, the structural equation model (SEM), based on partial least squares path modeling (PLS-PM) [63], demonstrates the relationships between the photoperiod, feeding regime, lipid metabolism genes, serum metabolites, and body composition. Data related to growth performance and feed utilization of juvenile rainbow trout were also obtained in this study and have been presented in a separate unpublished paper.

## 3. Results

### 3.1. Body Composition

**Figure 1:**
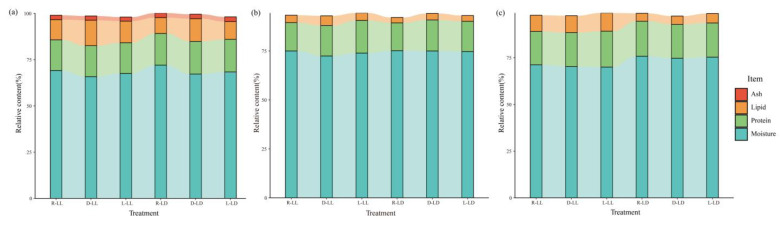
The body composition of juvenile *Oncorhynchus mykiss* under different experimental treatment. (**a**) Whole fish. (**b**) Liver. (**c**) Dorsal muscle.

The juvenile rainbow trout whole-body composition was influenced by the photoperiod and feeding regime (Figure 1a and Supplementary Table S3). The LL environment significantly increased whole-body lipid content and decreased whole-body ash content (*p* < 0.05). The whole-body moisture content in the R group was significantly higher than that in the L and D groups, and the whole-body lipid content in the D group was significantly higher than that in the R and L groups (*p* < 0.05). There was no interaction between the photoperiod and feeding regime on the whole-body composition (*p* > 0.05).

**Figure 4:**
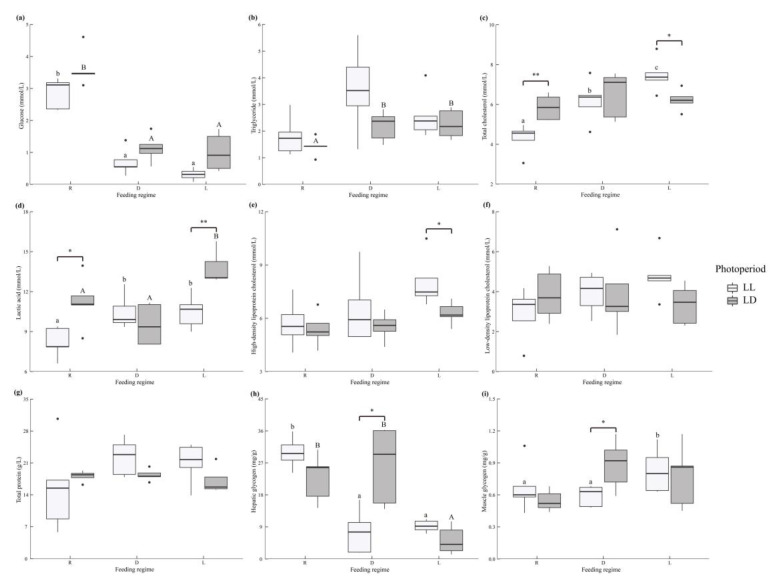
Serum metabolites and glycogen of juvenile *Oncorhynchus mykiss* under different experimental treatments (mean ± SD, *n* = 6). (**a**) Glucose; (**b**) triglyceride; (**c**) total cholesterol (**d**) lactic acid; (**e**) high-density lipoprotein cholesterol; (**f**) low-density lipoprotein cholesterol; (**g**) total protein; (**h**) hepatic glycogen; (**i**) muscle glycogen. Different lowercase letters and capital letters indicate a significant difference among the different feeding strategy at the LL (constant light) and LD (12L: 12D, lights-on at 6:00), respectively (*p* < 0.05). Asterisks denote significant differences between photoperiods at the same feeding strategy (* *p* < 0.05; ** *p* < 0.01).

The juvenile rainbow trout liver composition was influenced by the photoperiod and feeding regime (Figure 1b and Supplementary Table S3). The LL environment significantly reduced the liver moisture content and increased the liver lipid content (*p* < 0.05). The liver protein content in the R group was significantly lower than those in the L and D groups (*p* < 0.05). The liver glycogen content showed R > D > L (*p* < 0.05) (Figure 4h and Supplementary Table S3). Photoperiod and feeding regime had an interactive effect on the liver glycogen content (*p* < 0.05). In the LL environment, the R group liver glycogen content was significantly higher than the D and L groups (*p* < 0.05). In the LD environment, the L group liver glycogen content was significantly lower than the R and D groups (*p* < 0.05). Under the D treatment, the liver glycogen content of the LD group was significantly higher than that of the LL group.

The juvenile rainbow trout dorsal muscle composition was influenced by the photoperiod (Figure 1c and Supplementary Table S3). The LL environment significantly reduced the dorsal muscle moisture content and elevated the dorsal muscle lipid content (*p* < 0.05) and the dorsal muscle glycogen was significantly higher in the L group than in the R and D groups; in the D treatment, the dorsal muscle glycogen was markedly lower in the LL group than in the LD group (*p* < 0.05) (Figure 4i and Supplementary Table S3). There was no interaction between the photoperiod and feeding regime on the dorsal muscle composition (*p* > 0.05).

**Figure 2:**
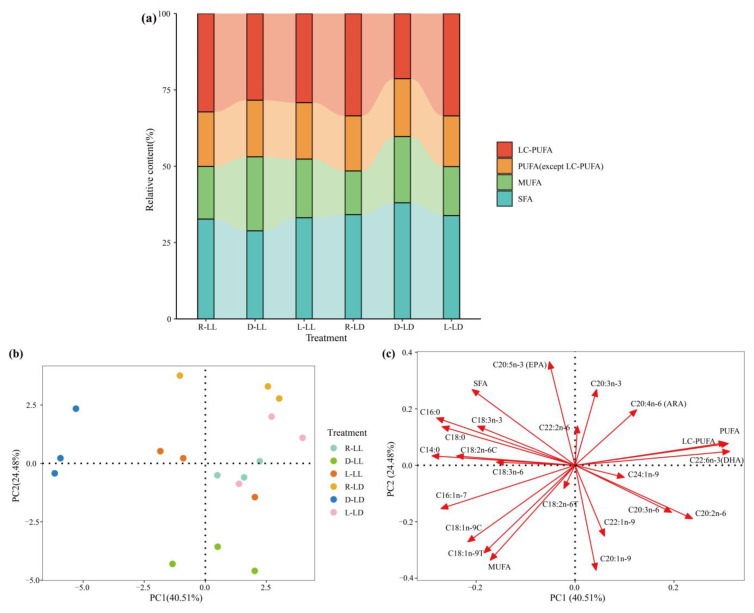

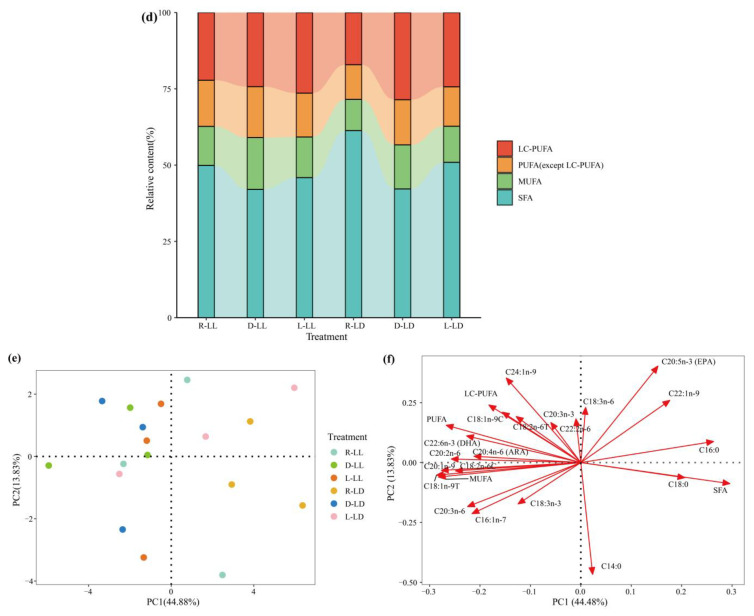

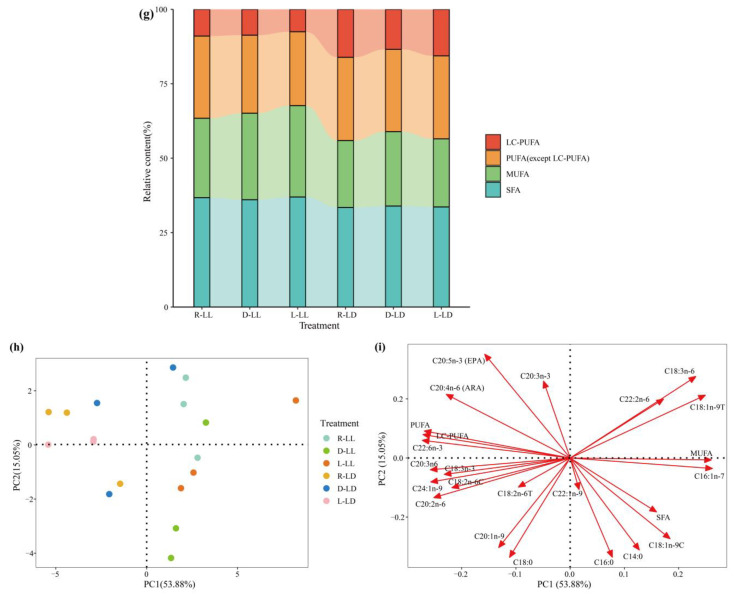
Fatty acids profile of juvenile *Oncorhynchus mykiss* under different experimental treatments. (**a**) Proportion of liver fatty acids; (**b**) PCA score plot of liver fatty acids; (**c**) PCA loading plot of liver fatty acids. (**d**) Proportion of serum fatty acids; (**e**) PCA score plot of serum fatty acids; (**f**) PCA loading plot of serum fatty acids. (**g**) Proportion of dorsal muscle fatty acids; (**h**) PCA score plot of dorsal muscle fatty acids; (**i**) PCA loading plot of dorsal muscle fatty acids.

The liver fatty acid profile was influenced by the photoperiod and feeding regime (Figure 2a and Supplementary Table S4). The LL environment significantly reduced the saturated fatty acid (SFA) proportion; the D group significantly increased the monounsaturated fatty acid (MUFA) proportion and significantly reduced the polyunsaturated fatty acid (PUFA) and long-chain polyunsaturated fatty acid (LC-PUFA) proportions compared to the R and L groups (*p* < 0.05). Photoperiod and feeding regime had an interactive effect on the liver SFA and PUFA proportions (*p* < 0.05). The results of the principal component analysis (PCA) revealed that the liver fatty acid profile in group D (D-LL and D-LD) was distinguished from the other groups (Figure 2b,c).

The serum fatty acids profile was mainly influenced by the feeding regime (Figure 2d and Supplementary Table S5). In the R group, the SFA proportion was significantly higher than that in the D group, the MUFA proportion was significantly lower than that in the D group, and the PUFA and LC-PUFA proportions were markedly lower than those in the D and L groups (*p* < 0.05). The PCA results revealed that the serum fatty acid profiles of the different treatments were not significantly separated (Figure 2e,f).

The dorsal muscle fatty acid profile was mainly influenced by the photoperiod (Figure 2g and Supplementary Table S6). The LL environment significantly increased the SFA and MUFA proportions and decreased the PUFA and LC-PUFA proportions (*p* < 0.05). The PCA results revealed that different photoperiods (LL vs. LD) were able to separate the fatty acid profiles of the dorsal muscle (Figure 2h,i).

### 3.2. Lipid Metabolism Genes

**Figure 3:**
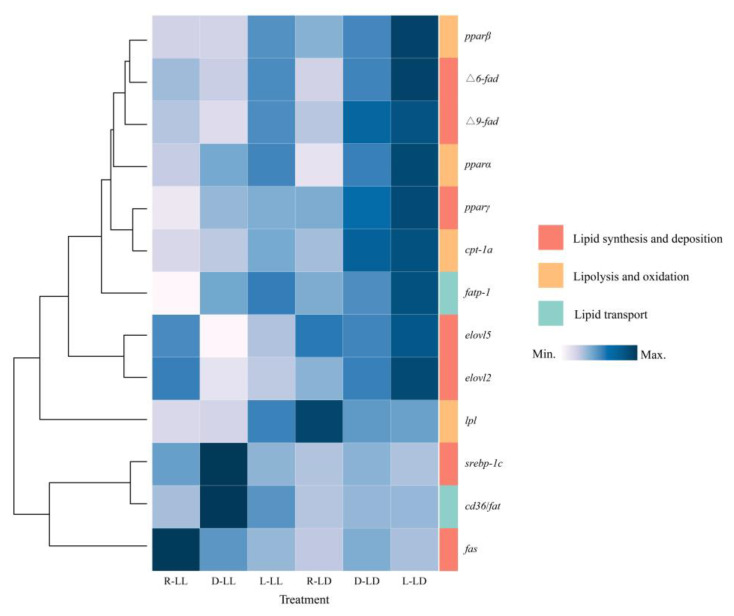
Gene expression of juvenile *Oncorhynchus mykiss* under different experimental treatments. *fas*: Fatty acid synthase, *Δ6-fad*: Δ6-fatty acid desaturase, *Δ9-fad*: Δ9-fatty acid desaturase, *elovl2*: Elongation of very long-chain fatty acid protein 2, *elovl5*: Elongation of very long-chain fatty acid protein 5, *srebp-1c*: Sterol regulatory element binding protein 1c, *pparα*: Peroxisome proliferators-activated receptor α, *pparβ*: Peroxisome proliferators-activated receptor β, *pparγ*: Peroxisome proliferators-activated receptor γ, *cpt-1a*: Carnitine palmitoyl transferase 1a, *lpl*: Lipoprotein lipase, *cd36*/*fat*: Cluster of differentiation 36/Fatty acid translocase, *fatp-1*: Fatty acid transport protein 1.

In the present study, lipid metabolism-related genes were influenced by the photoperiod and feeding regime (Figure 3 and Supplementary Table S7). Lipid metabolism genes were simply classified into three major classes according to their functions: lipid synthesis and deposition, lipolysis and oxidation, and lipid transport.

For lipid synthesis and deposition, the *fas*, *Δ9-fad*, *elovl2*, *elovl5*, *srebp-1c*, and *pparγ* genes’ expression were affected by the photoperiod (Figure 3 and Supplementary Table S7). The LL environment resulted in the upregulation of *fas* and *srebp-1c* gene expression and the downregulation of the expression of the *Δ9-fad*, *elovl2*, *elovl5*, and *pparγ* genes. The feeding regime affected the expression of the *fas*, *Δ6-fad*, *Δ9-fad*, *srebp-1c*, and *pparγ* genes. The L group upregulated *Δ6-fad* and *Δ9-fad* gene expression, the D group upregulated *srebp-1c* gene expression, and the R group upregulated *pparγ* gene expression. There was an interactive effect of photoperiod and feeding strategy on *fas*, *Δ6-fad*, *Δ9-fad,* and *elovl2* gene expression.

For lipolysis and oxidation, *pparβ*, *cpt-1a*, and *lpl* gene expression was affected by the photoperiod (Figure 3 and Supplementary Table S7). The LL environment downregulated the expression of these three genes. The feeding regime affected the expression of the *pparα*, *pparβ*, and *cpt-1a* genes. The R group downregulated *pparα* gene expression, the L group upregulated *pparβ* gene expression, and the R group downregulated *cpt-1a* gene expression. There was an interactive effect of photoperiod and feeding regime on *cpt-1a* and *lpl* gene expression.

For lipid transport, *cd36*/*fat* and *fatp-1* gene expression was influenced by the photoperiod (Figure 3 and Supplementary Table S7). The LL environment upregulated *cd36*/*fat* gene expression and downregulated *fatp-1* gene expression. The feeding regime affected the expression of the *cd36*/*fat* and *fatp-1* genes. The expression of the *cd36*/*fat* gene was upregulated in group D. The trend of *fatp-1* gene expression can be shown as R < D < L.

### 3.3. Serum Metabolites

Serum glucose (Glu), triglyceride (TG), total cholesterol (T-CHO), lactic acid (LA), and high-density lipoprotein cholesterol (H-DLC) were affected by the photoperiod and feeding regime (Figure 4a–e and Supplementary Table S3). The LL environment significantly reduced the LA levels (*p* < 0.05). The R group had a significantly higher Glu than the D and L groups, and the R group had significantly lower TG, LA, and H-DLC than the D group (*p* < 0.05). Individually, under the LD environment, TG was significantly lower in the R group than in the D and L groups. Under the LL environment, T-CHO showed R < D < L while under the L treatment, the LL environment significantly increased the H-DLC levels (*p* < 0.05).

### 3.4. Structural Equation Model

**Figure 5:**
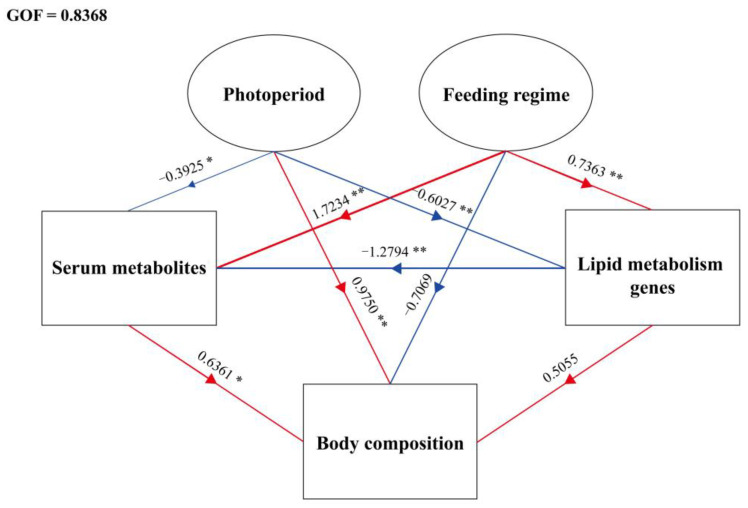
The structural equation model (SEM) based on partial least squares path modeling (PLS-PM) demonstrates the relationships between the photoperiod, feeding regime, digestive enzyme, lipid metabolism genes, serum hormones, serum metabolites, and body composition. Numbers on arrows are path coefficients. * means *p* < 0.05, ** means *p* < 0.01. Arrow widths show the strength of the causal relationship. Red arrows indicate positive correlation, blue arrows indicate negative correlation.

The structural equation model (SEM) based on partial least squares path modeling (PLS-PM) demonstrates the relationships between the photoperiod, feeding regime, lipid metabolism genes, serum metabolites, and body composition (Figure 5). The observed variables in this study were classified into the above five latent variables, and those with a loading value < 0.7 were excluded (Supplementary Figure S2) [63]. Photoperiod was significantly negatively correlated with lipid metabolism genes (path coefficients (PC) = −0.6027, *p* < 0.01) and serum metabolites (PC = −0.3925, *p* < 0.05), and was positively correlated with body composition (PC = 0.9750, *p* < 0.01). Feeding regime was significantly positively correlated with lipid metabolism genes (PC = 0.7363, *p* < 0.01) and serum metabolites (PC = 1.7234, *p* < 0.01), and negatively correlated with body composition (PC = −0.7069, *p* = 0.215). Lipid metabolism genes were significantly negatively correlated with serum metabolites (PC = −1.2794, *p* < 0.01) and lipid metabolism genes (PC = 0.5055, *p* = 0.294) and serum metabolites (PC = 0.6361, *p* < 0.05) were positively correlated with body composition. The goodness of fit (GOF) of the model was 0.8368.

## 4. Discussion

In the present study, LL decreased the moisture content in the liver and dorsal muscle and increased the lipid content in the whole-body, liver, and dorsal muscle. Similarly, LL increased the whole-body lipid content of Atlantic salmon (*Salmo salar*) [55]. In gibel carp (*Carassius auratus*), the lipid content in the whole-body, liver, and muscle gradually increased with prolonged light exposure [50]. An interesting phenomenon was observed that LL led to a decrease in the ash content of the whole-body. Some studies in Atlantic salmon have found that LL decreases the bone mineral content [64], affects mineralization, and delays osteoid incorporation [65], and even causes vertebrae malformations [66]. Similarly, LL caused a higher lower jaw malformation in European sea bass (*Dicentrarchus labrax*) [67]. In gilthead seabream (*Sparus aurata*), Mhalhel et al. (2020) [68] found that exogenous melatonin supplementation affected normal skeletogenesis and caused bone deformities by regulating the expression of genes related to bone formation. Fish receive external light signals and convert environmental time cues into endogenous biological signals through melatonin secretion [53]. Therefore, the increased bone deformity caused by LL may be related to the loss of coordination between the skeletal muscle and bone function caused by the disturbance of the circadian clock [68]. Although no bone deformities were observed in juvenile rainbow trout in the LL group, the significantly low whole-body ash and higher lipid deposition may be a warning sign of concern in the metabolic disturbance.

Aside from light, the feeding regime also affected the rainbow trout’s body composition. The R group fish had a higher whole-body moisture, lower whole-body lipid, and lower liver protein, which may reflect the poor nutritional status and feed utilization. This result is not surprising because random feeding could not induce food anticipatory activity (FAA) [69,70,71], thus failing to increase locomotor activity and optimize digestive and metabolic processes before feeding [72,73,74]. In addition, the serum Glu level in the R group was significantly higher than in the TRF group. Similar results have also been reported in gilthead seabream [74,75]. Sánchez et al. (2009) [75] concluded that random feeding stressed the fish, increasing the plasma Glu levels. The animal can store excess glucose in the form of glycogen in the liver and muscle [76]. In fish, hepatic glycogen synthesis is one of the metabolic pathways of blood glucose, and elevated blood glucose levels often cause a simultaneous increase in hepatic glycogen content [77]. Similarly, significantly higher liver glycogen was also observed in the R group. High hepatic glycogen levels reflect the passive adaptation of juvenile rainbow trout to high blood glucose. However, as a carnivorous fish, rainbow trout have a minimal ability to utilize glucose, so high glycogen levels may become a metabolic burden. On the other hand, random feeding may encourage fish to store more glycogen in response to possible food deficiencies.

However, contrary to expectations, TRF did not alleviate the whole-body, liver, and muscle lipid deposition caused by the LL. Fish body lipid content results from the balance between lipid synthesis and catabolism. Previous studies have attributed the elevated body lipid content to the following four conditions: (1) stable lipolysis and increased lipogenesis; (2) decreased lipolysis and increased lipogenesis; (3) decreased lipolysis and stable lipogenesis; and (4) slightly increased lipolysis and vastly increased lipogenesis [78]. The liver is the central organ of lipid metabolism, and the expression of lipid metabolism-related genes in the liver reflects the lipid metabolism of juvenile rainbow trout. In the present study, LL upregulated the expression of *fas* and *srebp-1c* genes and downregulated the expression of *pparα*, *pparβ*, *cpt-1a*, and *lpl* genes. Based on the above results, the rise in the lipid content of juvenile rainbow trout caused by LL may be due to (2) or (3). In addition, *cd36*/*fat* gene expression was significantly upregulated, and *fatp-1* gene expression was significantly downregulated in the LL group. Studies in mice have found that CD36/FAT protein promotes the intestinal absorption of fatty acids [21], and the upregulation of *cd36*/*fat* expression in the liver is associated with hepatic TG accumulation, elevated serum TG, and obesity [79]. In this study, the serum TG and H-DLC levels were also increased by LL. The *fatp-1* gene is involved in the uptake and oxidation of long-chain fatty acids [22] and its expression is upregulated in the liver by the upstream gene *pparα* [80]. The present study also observed the same expression trend of the *pparα* and *fatp-1* genes.

Notably, under the LL environment, TRF (especially in the L group) downregulated the mRNA abundance of *fas* and *srebp-1c* genes and upregulated the mRNA abundance of the *pparα*, *pparβ*, *cpt-1a*, *lpl*, and *fatp-1* genes. Similarly in mice livers, TRF was found to downregulate the expression of srebp-1c genes and upregulate the expression of *cpt-1a* genes [81]. Theoretically, lipid synthesis was inhibited in the TRF group, while oxidative catabolism was enhanced, which should lead to a decrease in lipid content. However, TRF also induced FAA (unpublished data), which led to an enhanced ability of rainbow trout to obtain lipids from the diet. In serum, the TG, T-CHO, and H-DLC levels in the TRF group were higher than those in the R group, reflecting a more active lipid metabolism and better nutritional status [82], thus supporting that the increased lipid absorption and transport level contributed to the body lipid deposition. Thus, in the RTF group, the uptake and utilization of exogenous lipids were probably prioritized over the synthesis and catabolism of endogenous lipids, which ultimately did not alleviate the lipid deposition caused by LL. On the other hand, although TRF corrected the attenuation of peripheral biological clock oscillations caused by LL [34] and alleviated the development of metabolic disease in a biological clock defect/metabolic disorder model in mammals [30,31], the relationship between the central and peripheral biological clocks in fish is more complex, and there does not seem to be a strict hierarchy between the central and peripheral biological clocks [83], which may be one of the reasons why TRF failed to alter the lipid deposition in the present study.

Though TRF did not alter the lipid deposition caused by LL, the resting period feeding still had a significantly different effect on the fish. The whole-body and the liver lipids were significantly higher in the D group than in the R and L groups in the LD photoperiod. In the wild, food is not continuously available, and fish exhibit different feeding rhythms to balance food availability and the occurrence of predators [84]. Fish metabolism is regulated by central and peripheral biological clocks (synchronized by light and food, respectively) [85]. Rainbow trout are diurnal feeders [86,87,88], and midnight feeding decouples the synchronization of the central and peripheral biological clocks, disrupts the metabolic process, and leads to lipid deposition and obesity [89]. Interestingly, it is noteworthy that the midnight feeding did not have the same effect in LL. This result suggests that the light–dark cycle may be a prerequisite for resting period feeding to induce lipid deposition, or that strict active and resting periods may not exist when light zeitgeber is absent. In the present study, compared with the L group, the D group had a higher expression of the *fas*, *srebp-1c*, and cd36/fat genes and a lower expression of the *pparα*, *pparβ*, and *cpt-1a* genes. These results suggest that lipid accumulation in the D group may be attributable to the upregulation of lipid synthesis and uptake. On the other hand, the upregulation of lipid oxidative catabolism in the L group may be associated with higher energy requirements for more frequent activity. The enhanced locomotor activity by TRF has also been widely reported in fishes [90,91]. In addition, higher muscle glycogen was observed in the L group. Rainbow trout muscles have been shown to have the ability to synthesize glycogen in situ [92], so high muscle glycogen may be an adaptation to the high energy demand of frequent locomotion in the L group. Muscle glycogen is not directly catabolized for energy but needs to be catabolized into LA and transported through the blood circulation to the liver for metabolism. A high serum LA level was detected in the L group.

Furthermore, this study also analyzed the fatty acid composition in the liver, serum, and muscle of juvenile rainbow trout. As the central organ of lipid metabolism, the liver reflects the metabolic process of fatty acid synthesis and decomposition. Fatty acids are the favored source of metabolic energy in fish [93]. Tocher (2003) [94] concluded that the priority of fatty acid oxidation for energy supply in most fish was SFA > MUFA > PUFA > LC-PUFA. In this study, the liver’s SFA (mainly C16:0) proportion was lower in the LL group than in the LD group, indicating an enhanced energy metabolism due to ALAN [95], as C16:0 is preferentially used for energy consumption when subjected to LL stress [96,97]. Furthermore, interestingly, the MUFA proportion in the LL group was higher than that in the LD group, but the PUFA and LC-PUFA proportions did not differ between the two photoperiods, suggesting that the excess MUFA in the LL group did not further synthesize PUFA and LC-PUFA. MUFA can be further synthesized into PUFA and LC-PUFA by elongase and desaturase [93]. The relative expression levels of the *Δ6-fad*, *Δ9-fad*, *elovl2*, and *elovl5* genes in the LL group were lower than those in the LD group. These results imply that LL may hinder the synthesis of PUFA and LC-PUFA by inhibiting the expression of elongase and desaturase genes. In Atlantic salmon, Nemova (2021) [54] found that LL increased the LC-PUFA (EPA and DHA) levels and 16:0/18:1n9 ratio, which was considered as preparation for smoltification. However, this phenomenon was not observed in the present study, probably because rainbow trout are landlocked salmonids.

Regarding the feeding regime, the TRF groups had higher MUFA and lower PUFA and LC-PUFA (especially in the D group) compared with the R group. The gene expression analysis of the PUFA-related synthases showed that the expression levels of the *Δ6-fad* and *Δ9-fad* genes in the TRF groups were higher than in the R group. In contrast, the feeding regime did not affect the relative expression of the *elovl2* and *elovl5* genes, implying that the expression patterns of the desaturase and elongase genes were different in rainbow trout. Furthermore, an interaction between the feeding regime and photoperiod on the fatty acid profile of rainbow trout liver was observed. Under the LD photoperiod, the proportion of PUFA was significantly lower in the D group than in the R and L groups. However, there was no statistical difference in the proportion of PUFA under the LL, and the proportion of PUFA in the D-LD group was also significantly lower than that in the D-LL group. First, compared with the L group, the expression levels of the *Δ6-fad*, *Δ9-fad*, *elovl2*, and *elovl5* genes were lower in the D group, indicating that mismatched feeding may inhibit the synthesis of PUFA in rainbow trout. Similarly, mismatched feeding resulted in the downregulation of the expression of the PUFA synthesis gene (*elovl6*) in darkbarbel catfish (*Pelteobagrus vachellii*) [98]. In addition, the cd36/fat gene expression was significantly higher in the D-LL group than in the D-LD group, indicating that LL promoted fatty acid uptake in the D-LL group. These phenomena eventually led to a significantly lower PUFA proportion observed only in the D-LD group. The results of liver fatty acid PCA analysis also clearly distinguished the D-LD group from the other groups.

The fatty acid profile in serum reflects the transport of fatty acids, and unsurprisingly, feeding regime caused extensive changes, with little contribution from the photoperiod. Briefly, the TRF groups had lower SFA and relatively higher MUFA, PUFA, and LC-PUFA than the R group. Additionally, a higher expression of the *fatp1* gene was detected in the TRF group. Compared with SFA, PUFA and LC-PUFA play a more important role in fish [93]. The present study results suggest that TRF facilitated the absorption and transport of these critical fatty acids and that FAA induced by TRF may play an important role in this process. In contrast to the serum, the fatty acid composition in muscle was mainly affected by the photoperiod and was almost independent of the feeding regime. Compared to the LD group, the LL group had a higher SFA, MUFA, and lower PUFA. A previous study suggested that highly unsaturated fatty acids (HUFA) (mainly EPA and DHA) have anti-stress effects in fish [99]. In the present study, LL exposure may have caused stress in rainbow trout [97], leading to a decrease in the proportion of HUFA in muscle. On the other hand, it was found that rainbow trout [100] and Mesopotamian catfish (*Silurus triostegus*) [101] had a higher MUFA and lower PUFA in summer (long daylight) than in winter (short daylight). Xie et al. (2013) [99] suggested that seasons affect fatty acids mainly related to changes in the photoperiod and temperature. The differential effects of photoperiod and feeding regime on the fatty acid profiles of different tissues reflect the functional variability between tissues. It has also been shown that the target tissues of light and food (as zeitgeber) are different in fish [90]. Further studies are needed to elucidate the molecular mechanisms underlying the differential effects of light and food on the fatty acid profiles of different tissues.

## 5. Conclusions

The present study investigated (1) the effects of ALAN on the body composition and lipid metabolism of juvenile rainbow trout (*Oncorhynchus mykiss*) through a LL environment and (2) whether the negative effects could be alleviated through TRF. The results showed that ALAN strongly impacted the lipid metabolism in juvenile rainbow trout, increased lipid synthesis, and decreased lipid oxidative catabolism, leading to lipid accumulation and a significant decrease in LC-PUFA proportion in the muscle. Unexpectedly, TRF could not alleviate rainbow trout lipid deposition caused by ALAN. Even subjective nocturnal feeding (D-LL) tended to exacerbate lipid deposition. Unlike the strict subordination in mammals, the independent relationship between the central and peripheral biological clocks in fish could be an important reason as to why TRF does not work. Further studies are needed to elucidate the mechanism behind this.

## Data Availability

The data presented in this study are not publicly available but are available upon request from the corresponding author.

## Supplementary Materials

The following supporting information can be downloaded at: https://www.mdpi.com/article/10.3390/metabo12100904/s1, Figure S1: Schematic diagram of the culture tank, the left side is the side view, and the right side is the top view with the top cover removed (1. Feeding port, 2. LED full-spectrum light, 3. Shading cloth, 4. Top cover, 5. Water outlet, 6. water outlet pipe, 7. water inlet pipe, 8. water inlet, 9. blue tank wall, 10. beam for fixing LED full spectrum light); Figure S2: Correlation between latent and observed variables. Numbers on arrows are loading values (The loading value < 0.7 are excluded). *fatp-1*: Fatty acid transport protein 1, *cpt-1a*: Carnitine palmitoyl transferase 1a, *pparβ*: Peroxisome proliferators-activated receptor β, *pparα*: Peroxisome proliferators-activated receptor α, *pparγ*: Peroxisome proliferators-activated receptor γ, *elovl2*: Elongation of very long-chain fatty acid protein 2, *Δ9-fad*: Δ9-fatty acid desaturase, *Δ6-fad*: Δ6-fatty acid desaturase, TP: Total protein, L-DLC: Low-density lipoprotein cholesterol, H-DLC: High-density lipoprotein cholesterol, T-CHO: Total cholesterol, TG: Triglyceride, Glu: Glucose, M-LCPUFA: Dorsal muscle long-chain polyunsaturated fatty acid, M-PUFA: Dorsal muscle polyunsaturated fatty acid, M-MUFA: Dorsal muscle monounsaturated fatty acid, M-Lipid: Dorsal muscle lipid, M-Moisture: Dorsal muscle moisture, L-Lipid, Liver lipid. Table S1: The main nutrients of the commercial diet used in this experiment; Table S2: The specific primers used for real-time PCR in this study; Table S3: Proximate composition of juvenile Oncorhynchus mykiss under different experimental treatment; Table S4: Liver fatty acids composition of juvenile Oncorhynchus mykiss under different experimental treatment; Table S5: Serum fatty acids composition of juvenile Oncorhynchus mykiss under different experimental treatment; Table S6: Dorsal muscle fatty acids composition of juvenile Oncorhynchus mykiss under different experimental treatment; Table S7: Gene expression of juvenile Oncorhynchus mykiss under different experimental treatment. References [102,103,104,105,106,107,108,109] cited in the supplementary materials.

## References

[1] Cinzano P., Falchi F., Elvidge C.D. (2001). The first World Atlas of the artificial night sky brightness. Mon. Not. R. Astron. Soc..

[2] Longcore T., Rich C. (2004). Ecological light pollution. Front. Ecol. Environ..

[3] Tancredi S., Urbano T., Vinceti M., Filippini T. (2022). Artificial light at night and risk of mental disorders: A systematic review. Sci. Total Environ..

[4] Foster R.G., Helfrich-Förster C. (2001). The regulation of circadian clocks by light in fruitflies and mice. Philos. Trans. R. Soc. B-Biol. Sci..

[5] Ma D., Li S.M., Molusky M.M., Lin J.D. (2012). Circadian autophagy rhythm: A link between clock and metabolism?. Trends Endocrinol. Metab..

[6] Lai K.Y., Sarkar C., Ni M.Y., Gallacher J., Webster C. (2020). Exposure to light at night (LAN) and risk of obesity: A systematic review and meta-analysis of observational studies. Environ. Res..

[7] Obayashi K., Yamagami Y., Kurumatani N., Saeki K. (2020). Bedroom lighting environment and incident diabetes mellitus: A longitudinal study of the HEIJO-KYO cohort. Sleep Med..

[8] Huang W.Y., Ramsey K.M., Marcheva B., Bass J. (2011). Circadian rhythms, sleep, and metabolism. J. Clin. Investig..

[9] Fleury G., Masis-Vargas A., Kalsbeek A. (2020). Metabolic Implications of Exposure to Light at Night: Lessons from Animal and Human Studies. Obesity.

[10] Watanabe T. (1982). Lipid nutrition in fish. Comp. Biochem. Physiol. Part B Comp. Biochem..

[11] Xu H.Y., Han T., Li X.Y., Wang J.T., Zheng P.Q., Yin F., Wang C.L. (2020). Effects of dietary lipid levels on survival, growth performance, and antioxidant ability of the early juvenile *Scylla paramamosain*. Aquaculture.

[12] Wilson R.P. (1994). Utilization of dietary carbohydrate by fish. Aquaculture.

[13] Stone D.A.J. (2003). Dietary carbohydrate utilization by fish. Rev. Fish. Sci..

[14] Greene D.H., Selivonchick D.P. (1987). Lipid metabolism in fish. Prog. Lipid Res..

[15] Turchini G.M., Francis D.S., Du Z.Y., Olsen R.E., Ringø E., Tocher D.R., Hardy R.W., Kaushik S.J. (2022). The lipids. Fish Nutrition.

[16] Shimano H. (2000). Sterol Regulatory Element-binding Protein-1 as a Dominant Transcription Factor for Gene Regulation of Lipogenic Enzymes in the Liver. Trends Cardiovasc. Med..

[17] Pai W.Y., Hsu C.C., Lai C.Y., Chang T.Z., Tsai Y.L., Her G.M. (2013). Cannabinoid receptor 1 promotes hepatic lipid accumulation and lipotoxicity through the induction of SREBP-1c expression in zebrafish. Transgenic Res..

[18] Nilsson-Ehle P., Garfinkel A.S., Schotz M.C. (1980). Lipolytic enzymes and plasma lipoprotein metabolism. Annu. Rev. Biochem..

[19] Wang L., Kaneko G., Takahashi S.I., Watabe S., Ushio H. (2015). Identification and gene expression profile analysis of a major type of lipoprotein lipase in adult medaka *Oryzias latipes*. Fish. Sci..

[20] Wang C.C., Si L.F., Li W.Y., Zheng J.L. (2019). A functional gene encoding carnitine palmitoyltransferase 1 and its transcriptional and kinetic regulation during fasting in large yellow croaker. Comp. Biochem. Physiol. B-Biochem. Mol. Biol..

[21] Nassir F., Wilson B., Han X.L., Gross R.W., Abumrad N.A. (2007). CD36 is important for fatty acid and cholesterol uptake by the proximal but not distal intestine. J. Biol. Chem..

[22] Stahl A. (2004). A current review of fatty acid transport proteins (SLC27). Pflug. Arch.-Eur. J. Physiol..

[23] Schoonjans K., Staels B., Auwerx J. (1996). Role of the peroxisome proliferator-activated receptor (PPAR) in mediating the effects of fibrates and fatty acids on gene expression. J. Lipid Res..

[24] Tsai M.L., Chen H.Y., Tseng M.C., Chang R.C. (2008). Cloning of peroxisorne proliferators activated receptors in the cobia (*Rachycentron canadum*) and their expression at different life-cycle stages under cage aquaculture. Gene.

[25] Panda S. (2016). Circadian physiology of metabolism. Science.

[26] Zeb F., Wu X.Y., Fatima S., Zaman M.H., Khan S.A., Safdar M., Alam I., Feng Q. (2021). Time-restricted feeding regulates molecular mechanisms with involvement of circadian rhythm to prevent metabolic diseases. Nutrition.

[27] Chaix A., Manoogian E.N.C., Melkani G.C., Panda S. (2019). Time-restricted eating to prevent and manage chronic metabolic diseases. Annu. Rev. Nutr..

[28] Hatori M., Vollmers C., Zarrinpar A., DiTacchio L., Bushong E.A., Gill S., Leblanc M., Chaix A., Joens M., Fitzpatrick J.A.J. (2012). Time-restricted feeding without reducing caloric intake prevents metabolic diseases in mice fed a high-fat diet. Cell Metab..

[29] Chaix A., Zarrinpar A., Miu P., Panda S. (2014). Time-restricted feeding is a preventative and therapeutic intervention against diverse nutritional challenges. Cell Metab..

[30] Ye Y.Q., Xu H.P., Xie Z.B., Wang L., Sun Y.N., Yang H.Y., Hu D.D., Mao Y.L. (2020). Time-restricted feeding reduces the detrimental effects of a high-fat diet, possibly by modulating the circadian rhythm of hepatic lipid metabolism and gut microbiota. Front. Nutr..

[31] Chaix A., Lin T., Le H.D., Chang M.W., Panda S. (2019). Time-restricted feeding prevents obesity and metabolic syndrome in mice lacking a circadian clock. Cell Metab..

[32] Villanueva J.E., Livelo C., Trujillo A.S., Chandran S., Woodworth B., Andrade L., Le H.D., Manor U., Panda S., Melkani G.C. (2019). Time-restricted feeding restores muscle function in Drosophila models of obesity and circadian-rhythm disruption. Nat. Commun..

[33] Che T.T., Yan C., Tian D.Y., Zhang X., Liu X.J., Wu Z.M. (2021). Time-restricted feeding improves blood glucose and insulin sensitivity in overweight patients with type 2 diabetes: A randomised controlled trial. Nutr. Metab..

[34] Yamamuro D., Takahashi M., Nagashima S., Wakabayashi T., Yamazaki H., Takei A., Takei S., Sakai K., Ebihara K., Iwasaki Y. (2020). Peripheral circadian rhythms in the liver and white adipose tissue of mice are attenuated by constant light and restored by time-restricted feeding. PLoS ONE.

[35] Flanagan A., Bechtold D.A., Pot G.K., Johnston J.D. (2021). Chrono-nutrition: From molecular and neuronal mechanisms to human epidemiology and timed feeding patterns. J. Neurochem..

[36] Kang J., Ratamess N.A., Faigenbaum A.D., Bush J.A., Beller N., Vargas A., Fardman B., Andriopoulos T. (2021). Effect of Time-Restricted Feeding on Anthropometric, Metabolic, and Fitness Parameters: A Systematic Review. J. Am. Coll. Nutr..

[37] Paredes J.F., López-Olmeda J.F., Martínez F.J., Sánchez-Vázquez F.J. (2015). Daily rhythms of lipid metabolic gene expression in zebra fish liver: Response to light/dark and feeding cycles. Chronobiol. Int..

[38] FAO (2020). The State of World Fisheries and Aquaculture 2020.

[39] Davies T.W., Duffy J.P., Bennie J., Gaston K.J. (2014). The nature, extent, and ecological implications of marine light pollution. Front. Ecol. Environ..

[40] Bolton D., Mayer-Pinto M., Clark G.F., Dafforn K.A., Brassil W.A., Becker A., Johnston E.L. (2017). Coastal urban lighting has ecological consequences for multiple trophic levels under the sea. Sci. Total Environ..

[41] Li D., Huang J., Zhou Q.M., Gu L., Sun Y.F., Zhang L., Yang Z. (2022). Artificial light pollution with different wavelengths at night interferes with development, reproduction, and antipredator defenses of *Daphnia magna*. Environ. Sci. Technol..

[42] Bassi A., Love O.P., Cooke S.J., Warriner T.R., Harris C.M., Madliger C.L. (2022). Effects of artificial light at night on fishes: A synthesis with future research priorities. Fish Fish..

[43] Becker A., Whitfield A.K., Cowley P.D., Järnegren J., Naesje T.F. (2013). Potential effects of artificial light associated with anthropogenic infrastructure on the abundance and foraging behaviour of estuary-associated fishes. J. Appl. Ecol..

[44] Pulgar J., Zeballos D., Vargas J., Aldana M., Manriquez P.H., Manriquez K., Quijón P.A., Widdicombe S., Anguita C., Quintanilla D. (2019). Endogenous cycles, activity patterns and energy expenditure of an intertidal fish is modified by artificial light pollution at night (ALAN). Environ. Pollut..

[45] Nelson T.R., Michel C.J., Gary M.P., Lehman B.M., Demetras N.J., Hammen J.J., Horn M.J. (2021). Effects of artificial lighting at night on predator density and salmonid predation. Trans. Am. Fish. Soc..

[46] Zapata M.J., Sullivan SM P., Gray S.M. (2019). Artificial lighting at night in estuaries-Implications from individuals to ecosystems. Estuaries Coasts.

[47] Brüning A., Hölker F., Franke S., Kleiner W., Kloas W. (2018). Influence of light intensity and spectral composition of artificial light at night on melatonin rhythm and mRNA expression of gonadotropins in roach Rutilus rutilus. Fish Physiol. Biochem..

[48] Khan Z.A., Labala R.K., Yumnamcha T., Devi S.D., Mondal G., Devi H.S., Rajiv C., Bharali R., Chattoraj A. (2018). Artificial Light at Night (ALAN), an alarm to ovarian physiology: A study of possible chronodisruption on zebrafish (*Danio rerio*). Sci. Total Environ..

[49] O’Connor J.J., Fobert E.K., Besson M., Jacob H., Lecchini D. (2019). Live fast, die young: Behavioural and physiological impacts of light pollution on a marine fish during larval recruitment. Mar. Pollut. Bull..

[50] Wei H., Cai W.J., Liu H.K., Han D., Zhu X.M., Yang Y.X., Jin J.Y., Xie S.Q. (2019). Effects of photoperiod on growth, lipid metabolism and oxidative stress of juvenile gibel carp (*Carassius auratus*). J. Photochem. Photobiol. B Biol..

[51] Basili D., Lutfi E., Falcinelli S., Balbuena-Pecino S., Navarro I., Bertolucci C., Capilla E., Carnevali O. (2020). Photoperiod Manipulation Affects Transcriptional Profile of Genes Related to Lipid Metabolism and Apoptosis in Zebrafish (*Danio rerio*) Larvae: Potential Roles of Gut Microbiota. Microb. Ecol..

[52] Kupprat F., Hölker F., Kloas W. (2020). Can skyglow reduce nocturnal melatonin concentrations in Eurasian perch?. Environ. Pollut..

[53] Mondal G., Devi S.D., Khan Z.A., Yumnamcha T., Rajiv C., Devi H.S., Chattoraj A. (2021). The influence of feeding on the daily rhythm of mRNA expression on melatonin bio-synthesizing enzyme genes and clock associated genes in the zebrafish (*Danio rerio*) gut. Biol. Rhythm. Res..

[54] Nemova N. (2021). Different photoperiod regimes affect the fatty acid profile of juvenile Atlantic salmon *Salmo salar* L.: Aquaculture and conservation approach to restoration of wild populations. J. Am. Oil Chem. Soc..

[55] Ytrestøyl T., Hjelle E., Kolarevic J., Takle H., Rebl A., Afanasyev S., Krasnov A., Brunsvik P., Terjesen B.F. (2022). Photoperiod in recirculation aquaculture systems and timing of seawater transfer affect seawater growth performance of Atlantic salmon (*Salmo salar*). J. World Aquac. Soc..

[56] FAO (2020). FAO Yearbook. Fishery and Aquaculture Statistics 2018.

[57] Polakof S., Míguez J.M., Soengas J.L. (2007). Daily changes in parameters of energy metabolism in liver, white muscle, and gills of rainbow trout: Dependence on feeding. Comp. Biochem. Physiol. A-Mol. Integr. Physiol..

[58] Hernández-Pérez J., Míguez J.M., Librán-Pérez M., Otero-Rodiño C., Naderi F., Soengas J.L., López-Patiño M.A. (2015). Daily rhythms in activity and mRNA abundance of enzymes involved in glucose and lipid metabolism in liver of rainbow trout, *Oncorhynchus mykiss*. Influ. Light Food Availab. Chronobiol. Int..

[59] Nisembaum L.G., Velarde E., Tinoco A.B., Azpeleta C., de Pedro N., Alonso-Gómez A.L., Delgado M.J., Isorna E. (2012). Light-dark cycle and feeding time differentially entrains the gut molecular clock of the goldfish (*Carassius auratus*). Chronobiol. Int..

[60] Cunniff P., AOAC (1995). Official methods of analysis of AOAC international. Official Analytical Chemists.

[61] Liu T., Xu H.Y., Han T., Wang J.T., Yin F., Wang C.L. (2021). Effect of dietary egg yolk lecithin levels on survival, growth, lipid metabolism, and antioxidant capacity of early juvenile green mud crab *Scylla paramamosain*. Aquaculture.

[62] Livak K.J., Schmittgen T.D. (2001). Analysis of relative gene expression data using real-time quantitative PCR and the 2^−ΔΔCT^ method. Methods.

[63] Sanchez G. (2013). PLS Path Modeling with R. Trowchez Editions. Berkeley. http://www.gastonsanchez.com/PLSPathModelingwithR.pdf.

[64] Fjelldal P.G., Nordgarden U., Berg A., Grotmol S., Totland G.K., Wargelius A., Hansen T. (2005). Vertebrae of the trunk and tail display different growth rates in response to photoperiod in Atlantic salmon, *Salmo salar* L., post-smolts. Aquaculture.

[65] Wargelius A., Fjelldal P.G., Nordgarden U., Hansen T. (2009). Continuous light affects mineralization and delays osteoid incorporation in vertebral bone of Atlantic salmon (*Salmo salar* L.). J. Exp. Biol..

[66] Fjelldal P.G., Lock E.J., Hansen T., Waagbo R., Wargelius A., Martens L.G., El-Mowafi A., Onsrud R. (2012). Continuous light induces bone resorption and affects vertebral morphology in Atlantic salmon (*Salmo salar* L.) fed a phosphorous deficient diet. Aquac. Nutr..

[67] Villamizar N., Garciá-Alcazar A., Sánchez-Vázquez F.J. (2009). Effect of light spectrum and photoperiod on the growth, development and survival of European sea bass (*Dicentrarchus labrax*) larvae. Aquaculture.

[68] Mhalhel K., Germanà A., Abbate F., Guerrera M.C., Levanti M., Laurà R., Montalbano G. (2020). The Effect of Orally Supplemented Melatonin on Larval Performance and Skeletal Deformities in Farmed Gilthead Seabream (*Sparus aurata*). Int. J. Mol. Sci..

[69] Mistlberger R.E. (1994). Circadian food-anticipatory activity: Formal models and physiological mechanisms. Neurosci. Biobehav. Rev..

[70] Reebs S.G., Gallant B.Y. (1997). Food-anticipatory activity as a cue for local enhancement in golden shiners (Pisces: Cyprinidae, *Notemigonus crysoleucas*). Ethology.

[71] Chen W.M., Purser G.J. (2001). The effect of feeding regime on growth, locomotor activity pattern and the development of food anticipatory activity in greenback flounder. J. Fish Biol..

[72] Stephan F.K. (2002). The “other” circadian system: Food as zeitgeber. J. Biol. Rhythm..

[73] Vera L.M., De Pedro N., Gomez-Milán E., Delgado M.J., Sánchez-Muros M.J., Madrid J.A., Sánchez-Vázquez F.J. (2007). Feeding entrainment of locomotor activity rhythms, digestive enzymes and neuroendocrine factors in goldfish. Physiol. Behav..

[74] Montoya A., López-Olmeda J.F., Yúfera M., Sánchez-Muros M.J., Sánchez-Vázquez F.J. (2010). Feeding time synchronises daily rhythms of behaviour and digestive physiology in gilthead seabream (*Sparus aurata*). Aquaculture.

[75] Sánchez J.A., López-Olmeda J.F., Blanco-Vives B., Sánchez-Vázquez F.J. (2009). Effects of feeding schedule on locomotor activity rhythms and stress response in sea bream. Physiol. Behav..

[76] Polakof S., Panserat S., Soengas J.L., Moon T.W. (2012). Glucose metabolism in fish: A review. J. Comp. Physiol. B-Biochem. Syst. Environ. Physiol..

[77] Capilla E., Medale F., Navarro I., Panserat S., Vachot C., Kaushik S., Gutierrez J. (2003). Muscle insulin binding and plasma levels in relation to liver glucokinase activity, glucose metabolism and dietary carbohydrates in rainbow trout. Regul. Pept..

[78] Gong Y., Chen W., Dong H., Zhu X., Yang Y., Jin J., Liu H., Xie S. (2017). Effects of food restriction on growth, body composition and gene expression related in regulation of lipid metabolism and food intake in grass carp. Aquaculture.

[79] Koonen D.P.Y., Jacobs R.L., Febbraio M., Young M.E., Soltys C.L.M., Ong H., Vance D.E., Dyck J.R.B. (2007). Increased hepatic CD36 expression contributes to dyslipidemia associated with diet-induced obesity. Diabetes.

[80] Frohnert B.I., Hui T.Y., Bernlohr D.A. (1999). Identification of a functional peroxisome proliferator-responsive element in the murine fatty acid transport protein gene. J. Biol. Chem..

[81] Vieira R.F.L., Munoz V.R., Junqueira R.L., de Oliveira F., Gaspar R.C., Nakandakari S.C.B.R., Costa S.D., Torsoni M.A., da Silva A.S.R., Cintra D.E. (2021). Time-restricted feeding combined with aerobic exercise training can prevent weight gain and improve metabolic disorders in mice fed a high-fat diet. J. Physiol..

[82] Wagner T., Congleton J.L. (2004). Blood chemistry correlates of nutritional condition, tissue damage, and stress in migrating juvenile chinook salmon (*Oncorhynchus tshawytscha*). Can. J. Fish. Aquat. Sci..

[83] Sánchez-Bretaño A., Gueguen M.M., Cano-Nicolau J., Kah O., Alonso-Gomez A.L., Delgado M.J., Isorna E. (2015). Anatomical distribution and daily profile of gper1b gene expression in brain and peripheral structures of goldfish (*Carassius auratus*). Chronobiol. Int..

[84] López-Olmeda J.F., Sánchez-Vázquez F.J., Kulczykowska E., Popek W., Kapoor B.G. (2010). Feeding Rhythms in Fish: From Behavioural to Molecular Approach. Biological Clock in Fish.

[85] Vera L.M., Negrini P., Zagatti C., Frigato E., Sanchez-Vazquez F.J., Bertolucci C. (2013). Light and feeding entrainment of the molecular circadian clock in a marine teleost (*Sparus aurata*). Chronobiol. Int..

[86] Iigo M., Tabata M. (1997). Circadian Rhythms of Locomotor Activity in the Rainbow Trout *Oncorhynchus mykiss*. Fish. Sci..

[87] Sánchez-Vázquez F.J., Tabata M. (1998). Circadian rhythms of demand-feeding and locomotor activity in rainbow trout. J. Fish Biol..

[88] Bolliet V., Aranda A., Boujard T. (2001). Demand-feeding rhythm in rainbow trout and European catfish: Synchronisation by photoperiod and food availability. Physiol. Behav..

[89] Salgado-Delgado R.C., Saderi N., Basualdo M.D., Guerrero-Vargas N.N., Escobar C., Buijs R.M. (2013). Shift Work or Food Intake during the Rest Phase Promotes Metabolic Disruption and Desynchrony of Liver Genes in Male Rats. PLoS ONE.

[90] López-Olmeda J.F. (2017). Nonphotic entrainment in fish. Comp. Biochem. Physiol..

[91] Fortes-Silva R., Do Valle S.V., Lopez-Olmeda J.F. (2018). Daily rhythms of swimming activity, synchronization to different feeding times and effects on anesthesia practice in an Amazon fish species (*Colossoma macropomum*). Chronobiol. Int..

[92] Frolow J., Milligan C.L. (2004). Hormonal regulation of glycogen metabolism in white muscle slices from rainbow trout (*Oncorhynchus mykiss* Walbaum). Am. J. Physiol.-Regul. Integr. Comp. Physiol..

[93] Sargent J.R., Tocher D.R., Bell J.G., Halver J.E., Hardy R.W. (2002). The Lipid. Fish Nutrition.

[94] Tocher D.R. (2003). Metabolism and functions of lipids and fatty acids in teleost fish. Rev. Fish. Sci..

[95] Hillyer K.E., Beale D.J., Shima J.S. (2021). Artificial light at night interacts with predatory threat to alter reef fish metabolite profiles. Sci. Total Environ..

[96] Henderson R.J., Sargent J.R., Hopkins C.C.E. (1984). Changes in the content and fatty acid composition of lipid in an isolated population of the capelin *Mallotus villosus* during sexual maturation and spawning. Mar. Biol..

[97] Valenzuela A., Rodríguez I., Schulz B., Cortés R., Acosta J., Campos V., Escobar-Aguirre S. (2022). Effects of continuous light (LD24:0) modulate the expression of lysozyme, mucin and peripheral blood cells in rainbow trout. Fishes.

[98] Qin C.J., Gong Q., Wen Z.Y., Zou Y.C., Yuan D.Y., Shao T., Li H.T. (2017). Comparative analysis of the liver transcriptome of *Pelteobagrus vachellii* with an alternative feeding time. Comp. Biochem. Physiol. D-Genom. Proteom..

[99] Xie D.Z., Wang S.Q., You C.H., Chen F., Zhang Q.H., Li Y.Y. (2013). Influencing factors and mechanisms on HUFA biosynthesis in teleosts. J. Fish. Sci. China.

[100] Calabretti A., Cateni F., Procida G., Favretto L.G. (2003). Influence of environmental temperature on composition of lipids in edible flesh of rainbow trout (*Oncorhynchus mykiss*). J. Sci. Food Agric..

[101] Cengiz E.I., Unlu E., Bashan M., Satar A., Uysal E. (2012). Effect of seasonal variations on the fatty acid composition of total lipid phospholipid and triacylglicerol in the dorsal muscle of Mesopotamian catfish (*Silurus triostegus* Heckel, 1843) in Tigris River (Turkey). Turk. J. Fish. Aquat. Sci..

[102] Skiba-Cassy S., Geurden I., Panserat S., Seiliez I. (2016). Dietary methionine imbalance alters the transcriptional regulation of genes involved in glucose, lipid and amino acid metabolism in the liver of rainbow trout (*Oncorhynchus mykiss*). Aquaculture.

[103] Song X.R., Marandel L., Skiba-Cassy S., Corraze G., Dupont-Nivet M., Quillet E., Geurden I., Panserat S. (2018). Regulation by dietary carbohydrates of intermediary metabolism in liver and muscle of two isogenic lines of rainbow trout. Front. Physiol..

[104] Kamalam B.S., Médale F., Larroquet L., Corraze G., Panserat S. (2013). Metabolism and fatty acid profile in fat and lean rainbow trout lines fed with vegetable oil: Effect of carbohydrates. PLoS ONE.

[105] Velasco C., Comesaña S., Conde-Sieira M., Míguez J.M., Soengas J.L. (2019). Effects of CCK-8 and GLP-1 on fatty acid sensing and food intake regulation in trout. J. Mol. Endocrinol..

[106] Conde-Sieira M., Capelli V., Álvarez-Otero R., Díaz-Rúa A., Velasco C., Comesaña S., López M., Soengas J.L. (2020). Hypothalamic AMPKα2 regulates liver energy metabolism in rainbow trout through vagal innervation. Am. J. Physiol-Reg. I..

[107] Sánchez-Gurmaches J., Cruz-Garcia L., Gutiérrez J., Navarro I. (2012). Adiponectin effects and gene expression in rainbow trout: An in vivo and in vitro approach. J. Exp. Biol..

[108] Naderi F., Míguez J.M., Soengas J.L., López-Patiño M.A. (2019). SIRT1 mediates the effect of stress on hypothalamic clock genes and food intake regulators in rainbow trout, *Oncorhynchus mykiss*. Comp. Biochem. Phys. A.

[109] Panserat S., Plagnes-Juan E., Gazzola E., Palma M., Magnoni L.J., Marandel L., Viegas I. (2020). Hepatic glycerol metabolism-related genes in carnivorous rainbow trout (*Oncorhynchus mykiss*): Insights into molecular characteristics, ontogenesis, and nutritional regulation. Front. Physiol..

