# Supplementary material for: Time-Restricted Feeding Could Not Reduce Rainbow Trout Lipid Deposition Induced by Artificial Night Light
Source: Metabolites. 2022 Sep 26;12(10):904. doi: 10.3390/metabo12100904 (PMC9606968; doi:10.3390/metabo12100904)
Supplement: Supplementary file 1 [file metabolites-12-00904-s001.zip › metabolites-1922906-supplementary.pdf]

Supplementary Table S1. The main nutrients of the commercial diet used in this experiment.

Items	Composition (%)
Crude protein	≥42.0
Crude lipid	≥10.0
Crude fiber	≤5.0
Ash	≤18.0
Moisture	≤12.0
Total phosphorus	≥1.0
Lysine	≥2.2

Supplementary Table S2. The specific primers used for real-time PCR in this study.

Gene	Sequence (5′ to 3′)	Reference
fas	F: TGATCTGAAGGCCCGTGTCA<br>R: GGGTGACGTTGCCGTGGTAT	[102]
Δ6-fad	F: AGGGTGCCTCTGCTAACTGG<br>R: TGGTGTTGGTGATGGTAGGG	[103]
Δ9-fad	F: GCCGTCCGAGGGTTCTTCTT<br>R: CTCTCCCCACAGGCACCAAG	[104]
elovl2	F: TGTGGTTTCCCCGTTGGATGCC<br>R: ACAGAGTGGCCATTTGGGCG	[103]
elovl5	F: GAACAGCTTCATCCATGTCC<br>R: TGACTGCACATATCGTCTGG	[103]
srebp-1c	F: GACAAGGTGGTCCAGTTGCT<br>R: CACACGTTAGTCCGCATCAC	[105]
pparα	F: CTGGAGCTGGATGACAGTGA<br>R: GGCAAGTTTTTGCAGCAGAT	[105]
pparβ	F: CTGGAGCTGGATGACAGTGA<br>R: GTCAGCCATCTTGTTGAGCA	[104]
pparγ	F: GACGGCGGGTCAGTACTTTA<br>R: ATGCTCTTGGCGAACTCTGT	[105]
cpt-1a	F: TCGATTTTCAAGGGTCTTCG<br>R: CACAACGATCAGCAAACTGG	[106]
lpl	F: TAATTGGCTGCAGAAAACAC<br>R: CGTCAGCAAACTCAAAGGT	[105]
cd36/fat	F: CCACTGAAGTTGAGCCATGA<br>R: TGCTAGACTCATGCCGTGTC	[105]
fatp-1	F: AGGAGAGAACGTCTCCACCA<br>R: CGCATCACAGTCAAATGTCC	[107]
β-actin	F: GATGGGCCAGAAAGACAGCTA<br>R: TCGTCCCAGTTGGTGACGAT	[108]
ef1α	F: TCCTCTTGGTCGTTTCGCTG<br>R: ACCCGAGGGACATCCTGTG	[109]

fas: Fatty acid synthase, *Δ6-fad*: Δ6-fatty acid desaturase, *Δ9-fad*: Δ9-fatty acid desaturase, *elovl2*: Elongation of very long-chain fatty acid protein 2, *elovl5*: Elongation of very long-chain fatty acid protein 5, *srebp-1c*: Sterol regulatory element binding protein 1c, *pparα*: Peroxisome proliferators-activated receptor α, *pparβ*: Peroxisome proliferators-activated receptor β, *pparγ*: Peroxisome proliferators-activated receptor γ, *cpt-1a*: Carnitine palmitoyl transferase 1a, *lpl*: Lipoprotein lipase, *cd36/fat*: Cluster of differentiation 36/ Fatty acid translocase, *fatp-1*: Fatty acid transport protein 1, *ef1α*: Elongation factor-1α.

Supplementary Table S3. Proximate composition of juvenile *Oncorhynchus mykiss* under different experimental treatment.

		Experimental treatment						Pooled	Two-way ANOVA		
		R-LL	D-LL	L-LL	R-LD	D-LD	L-LD	SEM [3]	P [1]	F [2]	P × F
Whole fish	Moisture (%)	69.31±1.26	65.80±2.47	67.53±1.45	72.03±2.52	67.23±0.46	68.37±1.67	0.59	NS	$P < 0.01$	NS
	Protein (%)	16.58±0.32	16.75±0.18	16.64±0.09	17.11±0.52	17.66±1.41	17.64±1.99	0.22	NS$^{\Delta}$	NS$^{\Delta}$	NS$^{\Delta}$
	Lipid (%)	10.91±1.40	13.76±2.66	11.66±1.61	8.60±0.86^{A}	12.25±0.86^{B}	9.58±0.41^{A}	0.51	$P < 0.05$	$P < 0.01$	NS
	Ash (%)	2.39±0.15	2.31±0.10	2.21±0.08	2.44±0.02	2.44±0.21	2.54±0.36	0.05	$P < 0.05^{\Delta}$	NS$^{\Delta}$	NS$^{\Delta}$
Liver	Moisture (%)	74.98±0.53^{c}	72.43±0.12^{a}	73.88±0.19^{b}	75.10±0.70	74.98±1.09	74.65±0.52	0.26	$P < 0.05^{\Delta}$	NS$^{\Delta}$	NS$^{\Delta}$
	Protein (%)	14.46±0.11	15.54±1.33	16.69±0.85	14.21±0.79^{A}	15.83±0.48^{B}	15.47±0.36^{B}	0.25	NS	$P < 0.01$	NS
	Lipid (%)	3.82±0.59	4.95±0.61	3.82±0.27	2.79±0.05^{A}	3.34±0.18^{B}	2.96±0.19^{A}	0.19	$P < 0.01^{\Delta}$	NS$^{\Delta}$	NS$^{\Delta}$
	Glycogen (mg/g)	29.90±4.38^{b}	7.61±6.19^{a*}	9.24±1.67^{a}	22.84±6.69^{B}	26.30±10.80^{B}	5.28±3.91^{A}	2.09	NS$^{\Delta}$	$P < 0.01^{\Delta}$	$P < 0.05^{\Delta}$
Dorsal muscle	Moisture (%)	71.21±1.38*	70.32±1.48**	70.02±1.29*	75.85±0.24	74.76±0.38	75.35±1.73	0.64	$P < 0.01$	NS	NS
	Protein (%)	17.87±1.17	18.15±1.46	19.20±1.10	18.71±0.94	18.12±0.26	18.28±1.14	0.24	NS	NS	NS
	Lipid (%)	8.70±1.62*	9.06±2.11*	9.77±2.12*	4.25±0.83	4.47±0.75	5.09±0.78	0.64	$P < 0.01$	NS	NS
	Glycogen (mg/g)	0.67±0.24^{a}	0.59±0.10^{a*}	0.83±0.21^{b}	0.55±0.10	0.88±0.23	0.78±0.29	0.04	NS	NS	NS
Serum	Glu (mmol/L)[4]	2.86±0.47^{b}	0.70±0.42^{a}	0.31±0.18^{a}	3.62±0.58^{B}	1.13±0.43^{A}	1.01±0.59^{A}	0.24	$P < 0.01$	$P < 0.01$	NS
	TG (mmol/L)[5]	1.81±0.74	3.56±1.60	2.59±0.89	1.42±0.34^{A}	2.19±0.56^{B}	2.26±0.54^{B}	0.19	$P < 0.05$	$P < 0.05$	NS
	T-CHO (mmol/L)[6]	4.29±0.74^{a**}	6.18±1.07^{b}	7.48±0.85^{c*}	5.86±0.63	6.50±1.16	6.22±0.52	0.23	NS	$P < 0.01$	$P < 0.01$
	LA (μmol/L)[7]	8.19±1.15^{a*}	10.48±1.30^{b}	10.51±1.28^{b**}	11.24±1.95^{A}	9.53±1.53^{A}	13.80±1.24^{B}	0.40	$P < 0.01$	$P < 0.01$	$P < 0.01$
	H-DLC (mmol/L)[8]	5.70±1.33	6.52±1.99	8.06±1.46*	5.39±0.95	5.53±0.78	6.29±0.64	0.27	$P < 0.05$	$P < 0.05$	NS
	L-DLC (mmol/L)[9]	2.90±1.32	3.93±1.01	4.81±1.19	3.83±1.24	3.92±2.00	3.36±0.99	0.25	NS	NS	NS
	TP (g/L)[10]	15.57±9.67	22.27±4.04	21.00±4.47	18.08±1.18	18.35±1.27	17.19±2.84	0.91	NS$^{\Delta}$	NS$^{\Delta}$	NS$^{\Delta}$

[1] P: Photoperiod. [2] F: Feeding regime. [3] Pooled SEM: standard error of mean. [4] Glu: Glucose. [5] TG: Triglyceride. [6] T-CHO: Total cholesterol. [7] LA: Lactic acid. [8] H-DLC: High-density lipoprotein cholesterol. [9] L-DLC: Low-density lipoprotein cholesterol. [10] TP: Total protein. Asterisks denote significant differences between photoperiods at the same feeding regime (*, $P < 0.05$; **, $P < 0.01$). Different lowercase letters and capital letters indicate significant difference among different feeding scheduled at the 24L:0D and 12L:12D photoperiod, respectively ($P < 0.05$). $^{\Delta}$ Mean that the Scheirer-Ray-Hare test was applied. NS: non-significant differences.

Supplementary Table S4. Liver fatty acids composition of juvenile *Oncorhynchus mykiss* under different experimental treatment (mean±SD, n=3)

Fatty acids	Experimental treatment						Pooled	Two-way ANOVA		
	R-LL	D-LL	L-LL	R-LD	D-LD	L-LD	SEM [3]	P [1]	F [2]	P × F
C14:0	0.88±0.09	0.78±0.10*	0.93±0.14	0.81±0.14^{A}	1.11±0.10^{B}	0.72±0.10^{A}	0.04	NS	NS	$P < 0.01$
C16:0	17.74±0.64	14.75±1.43**	17.60±2.26	18.06±2.24^{A}	23.02±0.18^{B}	15.34±1.41^{A}	0.72	$P < 0.05$	NS	$P < 0.01$
C18:0	7.96±0.13^{a*}	8.49±0.22^{ab**}	8.79±0.41^{b}	8.89±0.37^{A}	10.14±0.43^{B}	8.31±0.34^{A}	0.18	$P < 0.01$	$P < 0.01$	$P < 0.01$
∑SFA[4]	32.69±0.44	28.85±1.67**	33.14±2.77	34.14±3.65	38.02±0.09	33.84±1.04	0.77	$P < 0.01$	NS	$P < 0.01$
C16:1n-7	1.21±0.22	1.39±0.17	1.28±0.19	0.89±0.10^{A}	1.90±0.42^{B}	1.03±0.43^{A}	0.10	NS	$P < 0.01$	$P < 0.05$
C:181n-9T	11.85±0.55^{a}	17.85±0.33^{c*}	14.00±0.54^{b}	10.55±0.89^{A}	16.07±0.99^{B}	11.71±1.38^{A}	0.65	$P < 0.01$	$P < 0.01$	NS
C:181n-9C	1.27±0.16	1.47±0.07	1.28±0.05	1.07±0.03^{A}	1.48±0.09^{B}	1.10±0.12^{A}	0.05	$P < 0.05$	$P < 0.01$	NS
C20:1n-9	1.86±0.58	2.85±0.35**	1.93±0.54	1.08±0.12^{A}	1.54±0.05^{B}	1.63±0.09^{B}	0.15	$P < 0.05^{\Delta}$	NS$^{\Delta}$	NS$^{\Delta}$
C22:1n-9	0.08±0.03^{ab}	0.12±0.01^{b*}	0.04±0.04^{a}	0.07±0.02	0.06±0.02	0.06±0.04	0.01	NS	NS	NS
C24:1n-9	0.70±0.15	0.37±0.08	0.53±0.40	0.42±0.25	0.32±0.04	0.33±0.22	0.05	NS	NS	NS
∑MUFA[5]	17.25±0.63^{a*}	24.23±0.34^{c*}	19.20±0.16^{b}	14.31±0.91^{A}	21.69±1.51^{B}	16.03±2.20^{A}	0.85	NS$^{\Delta}$	$P < 0.01^{\Delta}$	NS$^{\Delta}$
C18:2n-6T	0.06±0.03	0.04±0.01	0.04±0.01	0.03±0.02	0.04±0.01	0.03±0.01	0.00	NS	NS	NS
C18:2n-6C	10.72±0.23	11.27±0.37	11.41±0.32**	11.17±0.79^{AB}	12.38±1.05^{B}	9.72±0.27^{A}	0.23	NS$^{\Delta}$	NS$^{\Delta}$	$P < 0.05^{\Delta}$
C18:3n-6	0.19±0.02	0.24±0.03	0.27±0.09	0.23±0.05	0.29±0.14	0.19±0.02	0.02	NS$^{\Delta}$	NS$^{\Delta}$	NS$^{\Delta}$
C18:3n-3	0.62±0.07	0.52±0.04**	0.63±0.08*	0.68±0.08^{B}	0.79±0.06^{C}	0.46±0.03^{A}	0.03	NS	$P < 0.05$	$P < 0.01$
C20:2n-6	3.26±0.13^{b}	3.35±0.18^{b**}	2.87±0.12^{a}	2.91±0.23	2.62±0.14	3.11±0.23	0.07	$P < 0.01$	NS	$P < 0.01$
C20:3n-6	2.88±0.06	3.00±0.15	3.10±0.13	2.81±0.23	2.67±0.02	2.95±0.10	0.04	$P < 0.05^{\Delta}$	NS$^{\Delta}$	NS$^{\Delta}$
C20:4n-6 (ARA)	4.41±0.14*	4.64±0.22	4.79±0.13	5.38±0.36^{B}	4.47±0.18^{A}	4.60±0.15^{A}	0.09	NS	NS	$P < 0.01$
C20:3n-3	0.07±0.01	0.06±0.02	0.08±0.04	0.12±0.02	0.09±0.07	0.11±0.05	0.01	NS	NS	NS
C20:5n-3 (EPA)	1.26±0.04^{c**}	0.97±0.04^{a**}	1.15±0.04^{b*}	1.46±0.05	1.42±0.14	1.29±0.07	0.04	$P < 0.01^{\Delta}$	NS$^{\Delta}$	NS$^{\Delta}$
C22:2n-6	0.06±0.05	0.06±0.03	0.07±0.01	0.10±0.04	0.06±0.02	0.04±0.01	0.01	NS	NS	NS
C22:6n-3 (DHA)	26.53±0.29	22.76±1.83**	23.26±2.83	26.68±3.14	15.45±0.45	27.64±1.10	1.07	NS$^{\Delta}$	$P < 0.05^{\Delta}$	NS$^{\Delta}$

∑PUFA[6]	50.06±0.54	46.92±1.92*	47.67±2.90	51.55±3.55^{B}	40.29±1.60^{A}	50.13±1.89^{B}	1.00	NS	$P < 0.01$	$P < 0.01$
∑LC-PUFA[7]	32.20±0.40	28.38±2.02**	29.20±2.95	33.51±3.41	21.33±0.56	33.52±1.29	1.11	NS$^{\Delta}$	$P < 0.05^{\Delta}$	NS$^{\Delta}$

[1] P: Photoperiod. [2] F: Feeding scheduled. [3] Pooled SEM: standard error of mean. [4] ∑SFA, saturated fatty acid: C4:0, C6:0, C8:0, C10:0, C11:0, C12:0, C13:0, C14:0, C15:0, C16:0, C17:0, C18:0, C20:0, C21:0, C22:0, C23:0, C24:0. [5] ∑MUFA, monounsaturated fatty acid: C14:1n-5, C15:1n-5, C16:1n-7, C17:1n-7, C18:1n-9T, C18:1n-9C, C20:1n-9, C22:1n-9, C24:1n-9. [6] ∑PUFA, poly unsaturated fatty acid: C18:2n-6T, C18:2n-6C, C18:3n-6, C18:3n-3, C20:2n-6, C20:3n-6, C20:4n-6, C20:3n-3, C20:5n-3, C22:2n-6, C22:6n-3. [7] ∑LC-PUFA, long-chain polyunsaturated fatty acid: C20:4n-6, C20:5n-3, C22:6n-3.

Asterisks denote significant differences between photoperiods at the same feeding scheduled (*, $P < 0.05$; **, $P < 0.01$).

Different lowercase letters and capital letters indicate significant difference among different feeding scheduled at the 24L:0D and 12L:12D photoperiod, respectively ($P < 0.05$).

$^{\Delta}$ Mean that the Scheirer-Ray-Hare test was applied.

Supplementary Table S5. Serum fatty acids composition of juvenile *Oncorhynchus mykiss* under different experimental treatment (mean±SD, n=3)

Fatty acids	Experimental treatment						Pooled	Two-way ANOVA		
	R-LL	D-LL	L-LL	R-LD	D-LD	L-LD	SEM [3]	P [1]	F [2]	P × F
C14:0	1.26±0.59	0.78±0.23	0.93±0.48	0.93±0.10	1.04±0.47	0.81±0.10	0.09	NS$^{\Delta}$	NS$^{\Delta}$	NS$^{\Delta}$
C16:0	19.66±0.29*	19.51±1.38	19.55±0.68	22.95±0.97^{B}	19.42±0.76^{A}	20.87±1.68^{AB}	0.37	*P* < 0.05	*P* < 0.05	NS
C18:0	10.54±1.49	9.57±0.96	10.07±0.57	10.26±1.37	9.09±0.56	10.35±1.61	0.26	NS	NS	NS
∑SFA[4]	49.89±6.32	42.04±4.95	45.90±1.74	61.31±4.41^{B}	42.19±3.64^{A}	50.91±8.36^{AB}	1.91	NS$^{\Delta}$	*P* < 0.05$^{\Delta}$	NS$^{\Delta}$
C16:1n-7	0.86±0.27	1.10±0.51	0.82±0.24	0.75±0.18	0.87±0.14	0.71±0.26	0.07	NS	NS	NS
C18:1n-9T	9.03±1.73	12.31±2.77	9.34±0.67	6.89±1.42	10.48±1.06	8.43±3.19	0.57	NS	*P* < 0.05	NS
C18:1n-9C	0.77±0.43	1.47±0.62	1.15±0.33	0.94±0.21	1.07±0.21	1.05±0.24	0.09	NS	NS	NS
C20:1n-9	0.75±0.14^{a}	1.17±0.15^{b}	1.04±0.09^{b}	0.63±0.14	1.04±0.09	0.66±0.42	0.06	NS	*P* < 0.05	NS
C22:1n-9	0.20±0.08	0.11±0.04	0.09±0.07	0.25±0.29	0.05±0.02	0.25±0.31	0.04	NS$^{\Delta}$	NS$^{\Delta}$	NS$^{\Delta}$
C24:1n-9	0.36±0.20	0.48±0.04	0.35±0.04	0.35±0.19	0.42±0.08	0.39±0.03	0.03	NS$^{\Delta}$	NS$^{\Delta}$	NS$^{\Delta}$
∑MUFA[5]	12.80±1.61	16.98±3.85	13.31±1.08	10.23±2.02	14.44±1.42	11.82±3.84	0.72	NS$^{\Delta}$	*P* < 0.05$^{\Delta}$	NS$^{\Delta}$
C18:2n-6T	0.29±0.15	0.36±0.22	0.24±0.11	0.25±0.14	0.27±0.06	0.16±0.02	0.03	NS$^{\Delta}$	NS$^{\Delta}$	NS$^{\Delta}$
C18:2n-6C	9.72±3.41	10.85±1.36	9.09±0.38	7.61±1.83	9.42±0.31	8.49±2.90	0.48	NS$^{\Delta}$	NS$^{\Delta}$	NS$^{\Delta}$
C18:3n-6	0.33±0.10	0.29±0.07	0.24±0.02	0.28±0.05	0.34±0.08	0.34±0.08	0.02	NS	NS	NS
C18:3n-3	0.84±0.35	0.77±0.14	0.80±0.33	0.80±0.19	0.76±0.09	0.65±0.28	0.05	NS	NS	NS
C20:2n-6	2.15±0.57	2.41±0.26*	2.11±0.07	1.28±0.14	1.94±0.10	1.80±0.49	0.11	*P* < 0.01	NS	NS
C20:3n-6	1.39±0.17	1.77±0.00	1.82±0.44	1.03±0.18	2.01±0.54	1.43±0.49	0.11	NS	*P* < 0.05	NS
C20:4n-6 (ARA)	2.20±0.25*	2.54±0.26	2.43±0.19*	1.70±0.15^{A}	3.28±0.55^{B}	1.93±0.25^{A}	0.14	NS	*P* < 0.01	*P* < 0.01
C20:3n-3	0.19±0.11	0.16±0.16	0.05±0.03	0.07±0.05	0.04±0.02	0.03±0.04	0.02	*P* < 0.05$^{\Delta}$	NS$^{\Delta}$	NS$^{\Delta}$
C20:5n-3 (EPA)	2.32±1.47	2.86±0.62	3.38±1.94	4.22±0.22	3.22±1.59	4.78±1.47	0.33	NS	NS	NS
C22:2n-6	0.22±0.21	0.07±0.01*	0.03±0.01	0.04±0.03	0.02±0.02	0.02±0.00	0.02	*P* < 0.05$^{\Delta}$	NS$^{\Delta}$	NS$^{\Delta}$
C22:6n-3 (DHA)	17.68±0.36^{a**}	18.91±0.77^{a}	20.60±1.17^{b}	11.17±0.99^{A}	22.07±2.51^{C}	17.65±2.50^{B}	0.89	NS$^{\Delta}$	*P* < 0.01$^{\Delta}$	NS$^{\Delta}$

∑PUFA[6]	37.31±4.74*	40.99±1.43	40.79±2.64	28.45±2.39^{A}	43.37±3.94^{B}	37.27±4.71^{B}	1.36	NS$^{\Delta}$	$P < 0.05^{\Delta}$	NS$^{\Delta}$
∑LC-PUFA[7]	22.19±1.16**	24.30±0.93	26.41±3.28	17.09±1.12^{A}	28.57±4.05^{B}	24.35±1.49^{B}	0.99	NS	$P < 0.01$	$P < 0.05$

[1] P: Photoperiod. [2] F: Feeding scheduled. [3] Pooled SEM: standard error of mean. [4] ∑SFA, saturated fatty acid: C4:0, C6:0, C8:0, C10:0, C11:0, C12:0, C13:0, C14:0, C15:0, C16:0, C17:0, C18:0, C20:0, C21:0, C22:0, C23:0, C24:0. [5] ∑MUFA, monounsaturated fatty acid: C14:1n-5, C15:1n-5, C16:1n-7, C17:1n-7, C18:1n-9T, C18:1n-9C, C20:1n-9, C22:1n-9, C24:1n-9. [6] ∑PUFA, poly unsaturated fatty acid: C18:2n-6T, C18:2n-6C, C18:3n-6, C18:3n-3, C20:2n-6, C20:3n-6, C20:4n-6, C20:3n-3, C20:5n-3, C22:2n-6, C22:6n-3. [7] ∑LC-PUFA, long-chain polyunsaturated fatty acid: C20:4n-6, C20:5n-3, C22:6n-3.

Asterisks denote significant differences between photoperiods at the same feeding scheduled (*, $P < 0.05$; **, $P < 0.01$).

Different lowercase letters and capital letters indicate significant difference among different feeding scheduled at the 24L:0D and 12L:12D photoperiod, respectively ($P < 0.05$).

$^{\Delta}$ Mean that the Scheirer-Ray-Hare test was applied.

Supplementary Table S6. Dorsal muscle fatty acids composition of juvenile *Oncorhynchus mykiss* under different experimental treatment (mean±SD, n=3)

Fatty acids	Experimental treatment						Pooled SEM [3]	Two-way ANOVA		
	R-LL	D-LL	L-LL	R-LD	D-LD	L-LD		P [1]	F [2]	P × F
C14:0	1.60±0.11	1.77±0.33	1.54±0.17	1.44±0.23	1.59±0.23	1.29±0.01	0.05	NS	NS	NS
C16:0	18.78±0.87	20.12±1.51	18.88±0.67*	18.61±1.39	19.72±1.05	17.59±0.23	0.29	NS	$P < 0.05$	NS
C18:0	5.71±0.16	6.10±0.26	5.92±0.38	6.19±0.39	5.87±0.25	6.12±0.10	0.07	NS	NS	NS
∑SFA[4]	35.04±1.26*	35.13±1.49	35.56±2.88	32.97±1.51	33.04±1.50	33.13±3.77	0.52	$P < 0.05$	NS	NS
C16:1n-7	2.99±0.20*	3.44±0.15	3.61±0.91	2.16±0.36	2.81±0.40	2.14±0.22	0.16	$P < 0.01$	NS	NS
C18:1n-9T	2.01±0.14**	1.42±0.88	2.24±1.57	0.51±0.30	1.25±0.97	0.45±0.18	0.24	$P < 0.05$	NS	NS
C18:1n-9C	18.02±0.89^{a}	20.56±0.78^{b**}	20.12±0.89^{b*}	17.27±0.49	17.56±0.37	17.82±0.83	0.34	$P < 0.01^{\Delta}$	NS$^{\Delta}$	NS$^{\Delta}$
C20:1n-9	0.64±0.03**	1.00±0.14	0.85±0.20	0.90±0.08	0.73±0.26	1.06±0.18	0.05	NS	NS	$P < 0.05$
C22:1n-9	0.14±0.02	0.13±0.01	0.15±0.11	0.18±0.04	0.12±0.03	0.07±0.05	0.01	NS$^{\Delta}$	NS$^{\Delta}$	NS$^{\Delta}$
C24:1n-9	0.27±0.04**	0.30±0.05	0.26±0.12	0.44±0.04	0.40±0.08	0.40±0.03	0.02	$P < 0.01$	NS	NS
∑MUFA[5]	26.02±0.87*	28.04±1.49	29.82±4.97	21.55±1.44	24.28±2.86	21.81±0.85	0.89	$P < 0.01$	NS	NS
C18:2n-6T	1.71±0.05	1.89±0.08*	1.89±0.20	1.80±0.23	1.66±0.05	2.15±0.43	0.06	NS	NS	NS
C18:2n-6C	18.00±0.58*	18.08±1.99	15.72±1.96	19.72±0.77	18.90±1.28	19.33±1.50	0.43	$P < 0.05$	NS	NS
C18:3n-6	2.97±0.33**	1.78±1.24	3.12±1.77	1.10±0.51	2.17±1.35	1.04±0.26	0.29	$P < 0.05^{\Delta}$	NS$^{\Delta}$	NS$^{\Delta}$
C18:3n-3	1.78±0.06**	1.73±0.24	1.44±0.22*	2.08±0.03	1.94±0.18	1.98±0.16	0.06	$P < 0.01$	NS	NS
C20:2n-6	1.09±0.05*	1.17±0.19	1.05±0.21*	1.41±0.19	1.19±0.26	1.50±0.14	0.05	$P < 0.05$	NS	NS
C20:3n-6	0.87±0.07*	0.93±0.08	0.76±0.24*	1.33±0.22	1.18±0.31	1.41±0.16	0.10	$P < 0.01$	NS	NS
C20:4n-6 (ARA)	1.25±0.06^{b*}	1.20±0.08^{b}	1.06±0.06^{a**}	1.49±0.13	1.49±0.24	1.44±0.06	0.05	$P < 0.01^{\Delta}$	NS$^{\Delta}$	NS$^{\Delta}$
C20:3n-3	0.13±0.04	0.12±0.01	0.13±0.02	0.15±0.03	0.12±0.05	0.13±0.02	0.01	NS	NS	NS
C20:5n-3 (EPA)	1.08±0.05*	1.00±0.17	0.98±0.06*	1.25±0.06	1.29±0.09	1.18±0.07	0.03	$P < 0.01$	NS	NS
C22:2n-6	1.05±0.45	0.51±0.17	0.73±0.09**	0.40±0.11	0.50±0.08	0.35±0.09	0.07	$P < 0.01^{\Delta}$	NS$^{\Delta}$	NS$^{\Delta}$
C22:6n-3 (DHA)	6.66±0.86*	6.48±0.58*	5.50±2.72*	13.38±2.34	10.64±2.47	13.01±1.47	0.87	$P < 0.01$	NS	NS

∑PUFA[6]	36.60±0.97*	34.90±1.10**	32.37±3.50*	44.09±3.01	41.08±1.71	43.51±3.33	1.18	$P < 0.01$	NS	NS
∑LC-PUFA[7]	8.99±0.93*	8.68±0.49*	7.53±2.74*	16.12±2.51	13.42±2.62	15.63±1.58	0.93	$P < 0.01$	NS	NS

[1] P: Photoperiod. [2] F: Feeding scheduled. [3] Pooled SEM: standard error of mean. [4] ∑SFA, saturated fatty acid: C4:0, C6:0, C8:0, C10:0, C11:0, C12:0, C13:0, C14:0, C15:0, C16:0, C17:0, C18:0, C20:0, C21:0, C22:0, C23:0, C24:0. [5] ∑MUFA, monounsaturated fatty acid: C14:1n-5, C15:1n-5, C16:1n-7, C17:1n-7, C18:1n-9T, C18:1n-9C, C20:1n-9, C22:1n-9, C24:1n-9. [6] ∑PUFA, poly unsaturated fatty acid: C18:2n-6T, C18:2n-6C, C18:3n-6, C18:3n-3, C20:2n-6, C20:3n-6, C20:4n-6, C20:3n-3, C20:5n-3, C22:2n-6, C22:6n-3. [7] ∑LC-PUFA, long-chain polyunsaturated fatty acid: C20:4n-6, C20:5n-3, C22:6n-3.

Asterisks denote significant differences between photoperiods at the same feeding scheduled (*, $P < 0.05$; **, $P < 0.01$).

Different lowercase letters and capital letters indicate significant difference among different feeding scheduled at the 24L:0D and 12L:12D photoperiod, respectively ($P < 0.05$).

[Δ] Mean that the Scheirer-Ray-Hare test was applied.

Supplementary Table S7. Gene expression of juvenile *Oncorhynchus mykiss* under different experimental treatment (mean±SD, n=6)

		Experimental treatment						Pooled SEM [3]	Two-way ANOVA		
		R-LL	D-LL	L-LL	R-LD	D-LD	L-LD		P [1]	F [2]	P × F
	fas	1.00±0.08^{c**}	0.50±0.09^{b}	0.38±0.09^{a}	0.29±0.12	0.42±0.19	0.34±0.09	0.04	P < 0.01	P < 0.01	P < 0.01
	Δ6-fad	1.02±0.22*	0.82±0.08**	1.44±0.46*	0.78±0.11^{A}	1.50±0.40^{B}	2.38±0.71^{C}	0.11	NS$^{\Delta}$	P < 0.01$^{\Delta}$	P < 0.05$^{\Delta}$
	Δ9-fad	1.04±0.30^{b}	0.54±0.20^{a**}	1.99±0.51^{c*}	1.00±0.35^{A}	2.79±0.71^{B}	3.38±1.16^{B}	0.20	P < 0.01	P < 0.01	P < 0.01
Lipid synthesis and deposition	*elovl2*	1.02±0.25^{c*}	0.29±0.09^{a**}	0.52±0.12^{b**}	0.71±0.19^{A}	1.02±0.22^{A}	1.61±0.36^{B}	0.08	P < 0.01$^{\Delta}$	NS$^{\Delta}$	P < 0.01$^{\Delta}$
	elovl5	1.03±0.27^{b}	0.61±0.14^{a}	0.83±0.17^{ab**}	1.08±0.38	1.04±0.43	1.29±0.29	0.06	P < 0.05$^{\Delta}$	NS$^{\Delta}$	NS$^{\Delta}$
	srebp-1c	1.02±0.22^{a**}	3.23±0.99^{b**}	0.68±0.11^{a**}	0.39±0.10^{A}	0.71±0.10^{B}	0.41±0.12^{A}	0.18	P < 0.01$^{\Delta}$	P < 0.01$^{\Delta}$	NS$^{\Delta}$
	pparγ	1.05±0.34^{a**}	2.03±0.48^{b*}	2.22±0.67^{b**}	2.25±0.38^{A}	3.24±0.87^{AB}	4.24±1.11^{B}	0.20	P < 0.01$^{\Delta}$	P < 0.01$^{\Delta}$	NS$^{\Delta}$
	pparα	1.01±0.19	1.20±0.44	1.33±0.51	0.89±0.25^{A}	1.35±0.47^{AB}	1.72±0.54^{B}	0.08	NS	P < 0.01	NS
Lipolysis and oxidation	*pparβ*	1.03±0.30^{a*}	1.02±0.28^{a**}	2.09±0.61^{b**}	1.64±0.40	2.21±0.47	3.72±0.66	0.17	P < 0.01$^{\Delta}$	P < 0.01$^{\Delta}$	NS$^{\Delta}$
	cpt-1a	1.03±0.28^{a**}	1.94±0.38^{b**}	3.78±1.13^{c**}	2.64±0.54^{A}	7.83±1.23^{B}	9.33±2.10^{B}	0.55	P < 0.01	P < 0.01	P < 0.01
	lpl	1.01±0.12^{a**}	1.06±0.18^{a**}	1.84±0.21^{b}	2.69±0.85^{B}	1.66±0.38^{A}	1.59±0.63^{A}	0.12	P < 0.01$^{\Delta}$	NS$^{\Delta}$	P < 0.01$^{\Delta}$
Lipid transport	*cd36/fat*	1.03±0.27^{a}	3.10±0.71^{b**}	1.56±0.57^{a}	0.94±0.23	1.15±0.35	1.14±0.18	0.14	P < 0.01	P < 0.01	P < 0.01
	fatp-1	1.08±0.46^{a**}	2.86±0.72^{b}	3.49±0.54^{b}	2.76±0.45^{A}	3.26±1.06^{A}	4.81±1.42^{B}	0.23	P < 0.05$^{\Delta}$	P < 0.01$^{\Delta}$	NS$^{\Delta}$

[1] P: Photoperiod. [2] F: Feeding scheduled. [3] Pooled SEM: standard error of mean. *fas*: Fatty acid synthase, *Δ6-fad*: Δ6-fatty acid desaturase, *Δ9-fad*: Δ9-fatty acid desaturase, *elovl2*: Elongation of very long-chain fatty acid protein 2, *elovl5*: Elongation of very long-chain fatty acid protein 5, *srebp-1c*: Sterol regulatory element binding protein 1c, *pparα*: Peroxisome proliferators-activated receptor α, *pparβ*: Peroxisome proliferators-activated receptor β, *pparγ*: Peroxisome proliferators-activated receptor γ, *cpt-1a*: Carnitine palmitoyl transferase 1a, *lpl*: Lipoprotein lipase, *cd36/fat*: Cluster of differentiation 36/ Fatty acid translocase, *fatp-1*: Fatty acid transport protein 1. NS: non-significant differences. Asterisks denote significant differences between photoperiods at the same feeding scheduled (*, P < 0.05; **, P < 0.01).

Different lowercase letters and capital letters indicate significant difference among different feeding scheduled at the 24L:0D and 12L:12D photoperiod, respectively (P < 0.05).

$^{\Delta}$ Mean that the Scheirer-Ray-Hare test was applied.

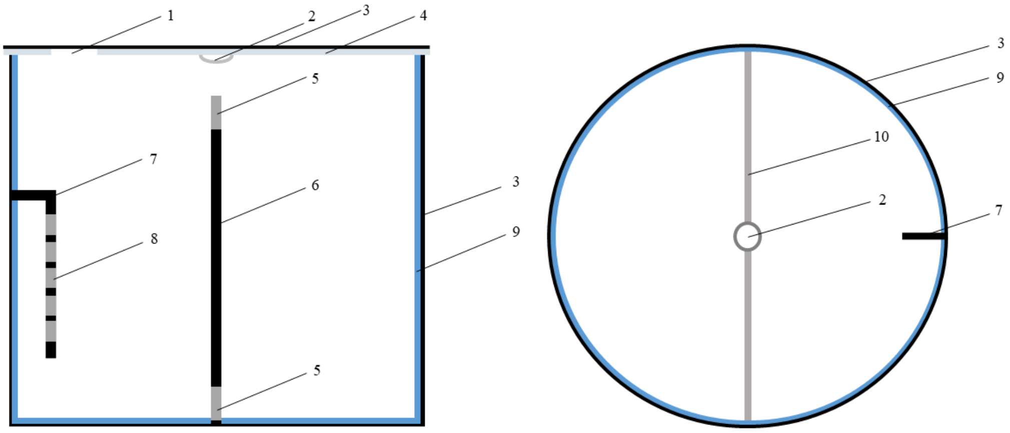

Supplementary Figure S1. Schematic diagram of the culture tank, the left side is the side view, and the right side is the top view with the top cover removed (1. Feeding port, 2. LED full-spectrum light, 3. Shading cloth, 4. Top cover, 5. Water outlet, 6. water outlet pipe, 7. water inlet pipe, 8. water inlet, 9. blue tank wall, 10. beam for fixing LED full spectrum light).

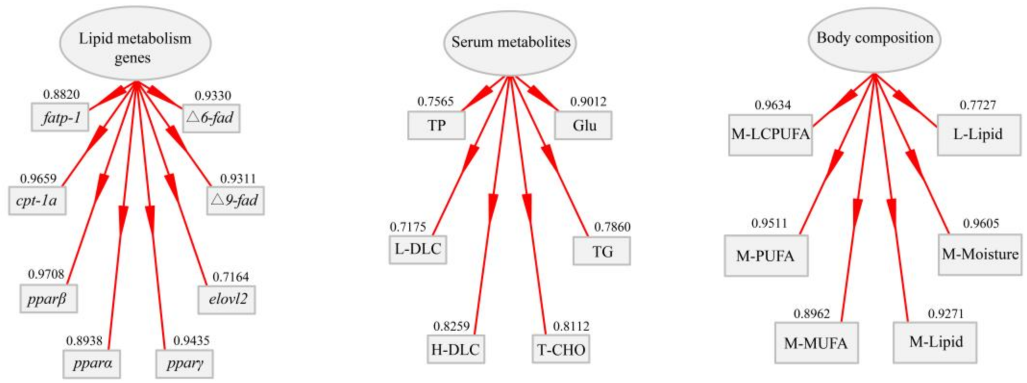

Supplementary Figure S2. Correlation between latent and observed variables. Numbers on arrows are loading values (The loading value < 0.7 are excluded). *fatp-1*: Fatty acid transport protein 1, *cpt-1a*: Carnitine palmitoyl transferase 1a, *pparβ*: Peroxisome proliferators-activated receptor *β*, *pparα*: Peroxisome proliferators-activated receptor *α*, *pparγ*: Peroxisome proliferators-activated receptor *γ*, *elovl2*: Elongation of very long-chain fatty acid protein 2, *Δ9-fad*: Δ9-fatty acid desaturase, *Δ6-fad*: Δ6-fatty acid desaturase, TP: Total protein, L-DLC: Low-density lipoprotein cholesterol, H-DLC: High-density lipoprotein cholesterol, T-CHO: Total cholesterol, TG: Triglyceride, Glu: Glucose, M-LCPUFA: Dorsal muscle long-chain polyunsaturated fatty acid, M-PUFA: Dorsal muscle polyunsaturated fatty acid, M-MUFA: Dorsal muscle monounsaturated fatty acid, M-Lipid: Dorsal muscle lipid, M-Moisture: Dorsal muscle moisture, L-Lipid, Liver lipid.
